# When Exercise ECG and Stress Echocardiography Disagree: Thoracic Geometry as a Potential Determinant of Exercise-Induced ST-Segment Abnormalities

**DOI:** 10.3390/jcm15218292

**Published:** 2026-10-24

**Authors:** Andrea Sonaglioni, Michele Lombardo, Gian Luigi Nicolosi, Massimo Baravelli, Marijana Tadic

**Affiliations:** 1 Division of Cardiology, IRCCS MultiMedica, 20123 Milan, Italy; michele.lombardo@multimedica.it (M.L.); massimo.baravelli@multimedica.it (M.B.); 2 Division of Cardiology, Policlinico San Giorgio, 33170 Pordenone, Italy; gianluigi.nicolosi@gmail.com; 3 Klinik für Innere Medizin II, Universitätsklinikum Ulm, 89081 Ulm, Germany; marijana_tadic@hotmail.com

**Keywords:** antero-posterior thoracic diameter, coronary artery disease, exercise electrocardiography, exercise stress echocardiography, mitral valve prolapse, thoracic geometry

## Abstract

**Background:** Exercise-induced ST-segment depression may occur despite negative exercise stress echocardiography (ESE) and absence of obstructive coronary artery disease (CAD), creating diagnostic uncertainty in symptomatic patients. Whether thoracic geometry contributes to this discordant response remains poorly understood. We investigated factors associated with a positive exercise ECG despite negative ESE (ECG+/ESE−), with particular focus on antero-posterior (A-P) thoracic diameter. **Methods:** In this prospective, single-center study, 101 consecutive symptomatic patients undergoing evaluation for suspected obstructive CAD were enrolled. ESE and coronary computed tomography angiography (CCTA) were prospectively planned in all patients. A positive exercise ECG was defined by horizontal/downsloping ST-segment depression ≥ 1.0 mm or upsloping depression ≥ 1.5 mm. ESE was considered negative in the absence of new or worsening regional wall-motion abnormalities. Patients with ECG+/ESE− and ECG−/ESE− responses were compared, and factors associated with ECG positivity were assessed by logistic regression. The discriminatory performance of A-P thoracic diameter was explored using ROC analysis. **Results:** All patients had negative ESE and no obstructive CAD on CCTA, while 35 (34.7%) developed a positive exercise ECG. Compared with ECG−/ESE− patients, those with ECG+/ESE− exhibited a smaller A-P thoracic diameter and higher modified Haller index, together with distinctive cardiac geometry and more frequent MVP. After multivariable adjustment, MVP was independently associated with ECG positivity (adjusted OR 8.50, 95% CI 1.07–67.75; *p* = 0.043), whereas increasing A-P thoracic diameter showed a strong inverse association (adjusted OR per 1-cm increase 0.026, 95% CI 0.005–0.143; *p* < 0.001). Smaller A-P thoracic diameter was also associated with lower exercise capacity and chronotropic response, greater exercise-induced ST-segment depression, and lower peak-exercise E/e′. A-P thoracic diameter showed excellent discrimination (AUC 0.962, 95% CI 0.921–1.000; *p* < 0.001); a data-derived cut-off ≤ 10.95 cm yielded 97.1% sensitivity and 93.9% specificity. **Conclusions:** MVP and reduced A-P thoracic diameter were independently associated with exercise ECG positivity despite negative ESE and absence of obstructive CAD, supporting a distinct thoraco-cardiac phenotype associated with exercise-induced repolarization abnormalities despite preserved LV contractile reserve. A-P thoracic diameter may provide complementary phenotypic information when exercise ECG and ESE findings are discordant; however, its incremental clinical value and the proposed ≤ 10.95-cm threshold require external validation.

## 1. Introduction

Coronary artery disease (CAD) remains a major cause of cardiovascular morbidity and mortality [1,2]. Its evaluation in symptomatic patients requires integration of clinical likelihood with anatomical and functional testing [3,4,5]. Contemporary European guidelines recommend estimating the likelihood of obstructive CAD on the basis of symptoms, age, sex, and cardiovascular risk factors, with subsequent diagnostic testing tailored to the individual clinical profile [6]. Within this framework, exercise testing remains clinically informative because it provides an integrated assessment of functional capacity, symptom reproduction, hemodynamic response, exercise-induced arrhythmias, and dynamic electrocardiographic changes [7]. Exercise stress echocardiography (ESE) further complements this information by directly evaluating inducible left ventricular (LV) regional wall-motion abnormalities and generally provides greater diagnostic specificity for obstructive CAD than exercise electrocardiography (ECG) alone [8,9,10].

Interpretation becomes more challenging when the electrocardiographic and echocardiographic responses to exercise are discordant. In clinical practice, exercise-induced ST-segment depression may occur despite preserved or hyperdynamic LV wall motion during stress, resulting in an ECG-positive/ESE-negative (ECG+/ESE−) phenotype [11,12,13]. Such discordance may generate diagnostic uncertainty because the ECG suggests myocardial ischemia whereas stress imaging provides no evidence of inducible regional contractile dysfunction. Consequently, clinicians may face uncertainty regarding whether the electrocardiographic abnormality reflects occult coronary disease, a non-obstructive ischemic mechanism, or a non-ischemic alteration in myocardial repolarization. This distinction is clinically relevant, as an abnormal exercise ECG may prompt additional anatomical or functional testing even when stress echocardiographic findings are reassuring.

The mechanisms underlying ECG+/ESE− discordance are likely heterogeneous. Exercise-induced ST-segment depression does not exclusively reflect flow-limiting epicardial coronary stenosis and may occur in association with coronary microvascular dysfunction [14], abnormal coronary vasomotion [15], or transient imbalance between myocardial oxygen supply and demand [16]. Patient-related and hemodynamic factors may further influence exercise-induced repolarization. Female sex [17,18], low clinical likelihood of obstructive CAD [19], an exaggerated hypertensive response to exercise [20,21,22,23], and hyperadrenergic activation [24] have all been associated with apparently false-positive or discordant stress-test findings. Hyperventilation represents another potential mechanism, particularly in individuals with mitral valve prolapse (MVP), in whom dynamic ST-segment abnormalities may occur in the absence of obstructive epicardial CAD [25]. Collectively, these observations emphasize that a positive exercise ECG should not automatically be considered synonymous with obstructive CAD and suggest that additional cardiovascular and extracardiac determinants may contribute to exercise-induced repolarization abnormalities.

Among these potential determinants, chest wall conformation has received comparatively limited attention. The anatomical relationship between the thoracic cage and the heart varies considerably among individuals and may influence cardiac position, chamber geometry, and the mechanical interaction between the heart and surrounding structures. A narrow or concave chest wall is characterized by a reduced antero-posterior (A-P) thoracic diameter and has been associated with a recognizable cardiovascular phenotype that includes MVP, atypical cardiovascular symptoms, ventricular ectopy, and exercise-induced ST-segment abnormalities despite a relatively low probability of obstructive CAD [26]. These observations raise the possibility that some exercise ECG abnormalities may reflect a thoraco-cardiac mechanical phenotype rather than conventional myocardial ischemia caused by obstructive epicardial disease.

Thoracic geometry can be characterized noninvasively during routine cardiovascular assessment. In particular, the modified Haller index (MHI), which incorporates the A-P thoracic diameter into a non-radiological measure of chest wall configuration, provides a simple means of identifying individuals with a relatively narrow or concave thorax [27]. Previous evidence has shown an inverse relationship between MHI and true-positive ESE findings and obstructive CAD [28], suggesting that chest wall configuration may influence the interpretation of exercise-induced electrocardiographic abnormalities. The A-P thoracic diameter itself may be particularly attractive in clinical practice because it represents a direct anatomical measurement that can be readily obtained from the parasternal long-axis echocardiographic view without additional imaging procedures or ionizing radiation. However, its specific relationship with exercise ECG positivity in patients with negative ESE has not been systematically characterized.

Clarifying this relationship could have practical implications for patients in whom exercise ECG and stress echocardiography provide apparently conflicting information. Identification of a simple extracardiac marker associated with ECG+/ESE− discordance could improve phenotypic interpretation of stress-test findings and generate a rationale for more individualized assessment of patients with suspected CAD.

Accordingly, we investigated clinical, anthropometric, electrocardiographic, hemodynamic, and echocardiographic factors independently associated with ECG+/ESE− in symptomatic patients evaluated for suspected obstructive CAD, with particular focus on A-P thoracic diameter. We hypothesized that a reduced A-P thoracic diameter would be independently associated with exercise-induced ST-segment depression despite negative ESE and absence of obstructive CAD. We also assessed the discriminatory performance of A-P thoracic diameter and explored the optimal threshold associated with exercise ECG positivity.

## 2. Materials and Methods

### 2.1. Study Design and Population

This prospective, observational, single-center study was conducted at the Cardiology Department of IRCCS MultiMedica–Ospedale San Giuseppe, Milan, Italy, within the framework of the prospective MVP&ESE project (Supplementary Material File S1). The original MVP&ESE protocol was designed to investigate the relationships among MVP, chest wall conformation, and cardiovascular response to exercise in symptomatic individuals undergoing ESE. The present analysis specifically focused on the discordance between exercise ECG and stress echocardiographic findings and on the potential contribution of thoracic geometry to this phenotype.

The study was conducted in accordance with the principles of the Declaration of Helsinki and its subsequent amendments. The study protocol was reviewed and approved by the Comitato Etico Territoriale Lombardia 5, with final approval issued on 3 November 2025 (Supplementary Material File S2), and all participants provided written informed consent before enrollment.

Consecutive adult patients referred to our Cardiology Outpatient Clinic for cardiovascular evaluation because of chest pain suggestive of angina, exertional and/or resting dyspnea, and/or palpitations were prospectively screened for eligibility. The present analysis included symptomatic individuals undergoing evaluation for suspected obstructive coronary artery disease (CAD) with a low-to-intermediate clinical likelihood according to the 2024 European Society of Cardiology Guidelines [6]. To permit direct comparison between functional and anatomical assessment, both ESE and coronary computed tomography angiography (CCTA) were prospectively planned as part of the diagnostic work-up. Eligibility was independent of the chronological order in which ESE and CCTA were performed and of the center at which CCTA was acquired.

Additional inclusion criteria were age ≥ 18 years, sinus rhythm and an interpretable resting 12-lead ECG allowing reliable assessment of exercise-induced ST-segment changes, ability to perform symptom-limited bicycle exercise, adequate transthoracic echocardiographic acoustic windows for assessment of regional LV wall motion at rest and during exercise, and availability of complete diagnostic ESE and CCTA data. Patients fulfilling these criteria were enrolled consecutively irrespective of the presence or absence of MVP or of their chest-wall configuration. MVP and thoracic geometry were prospectively assessed in all participants as prespecified phenotypic variables of the parent MVP&ESE project and did not constitute criteria for preferential recruitment or patient selection.

Patients were excluded in the presence of known obstructive CAD, previous myocardial infarction or coronary revascularization, hemodynamic instability, severe primary mitral regurgitation (MR), more-than-mild aortic stenosis or regurgitation, conditions precluding adequate physical exercise, inadequate echocardiographic acoustic windows, non-diagnostic ESE, or unavailable or non-diagnostic CCTA. Patients with baseline ECG abnormalities preventing reliable interpretation of exercise-induced ST-segment changes were also excluded from the present analysis.

CCTA served as the anatomical reference for excluding obstructive epicardial CAD. Obstructive CAD was defined as ≥50% luminal stenosis in at least one major epicardial coronary artery, according to contemporary guideline criteria [6]. Only patients without obstructive CAD were retained for the present analysis. Accordingly, individuals with completely normal coronary arteries and those with mild non-obstructive coronary atherosclerosis and/or calcification were eligible, whereas patients with ≥50% coronary stenosis were excluded. This approach was intended to specifically investigate exercise-induced electrocardiographic abnormalities occurring in the absence of flow-limiting epicardial coronary disease and to minimize the possibility that an ECG+/ESE− response merely reflected unrecognized obstructive CAD.

### 2.2. Clinical, Anthropometric, Resting Echocardiographic, and Carotid Ultrasound Assessment

At baseline, a comprehensive clinical evaluation was performed in all participants. Demographic characteristics, cardiovascular risk factors, relevant medical history, ongoing pharmacological treatment, and presenting symptoms were systematically recorded. Resting systolic and diastolic blood pressure, heart rate, and peripheral oxygen saturation were measured, and a standard 12-lead ECG was obtained to assess cardiac rhythm, heart rate, atrioventricular and intraventricular conduction, and baseline repolarization abnormalities. Laboratory assessment included hemoglobin, fasting glucose, serum creatinine, and low-density lipoprotein cholesterol. Body surface area (BSA) and body mass index (BMI) were calculated using standard formulas.

Resting transthoracic echocardiography (TTE) was performed with participants in the left lateral decubitus position using a Philips Sparq ultrasound system (Philips Healthcare, Andover, MA, USA). All examinations were performed by the same experienced cardiologist according to contemporary recommendations for cardiac chamber quantification [29,30] and assessment of LV diastolic function [31]. The prespecified echocardiographic protocol included interventricular septal and posterior wall thickness, LV end-diastolic diameter, relative wall thickness, LV mass indexed to BSA, LV geometry, LV ejection fraction (LVEF), transmitral E/A ratio, average E/e′ ratio, left atrial (LA) A-P diameter, right ventricular inflow tract dimension, tricuspid annular plane systolic excursion, and estimated systolic pulmonary artery pressure. Valvular morphology and function were systematically assessed according to contemporary recommendations [32,33].

Particular attention was paid to thoracic geometry, which was prospectively assessed as part of the MVP&ESE protocol. Chest wall conformation was characterized using two complementary measurements: the maximum latero-lateral (L-L) thoracic diameter and the minimum A-P thoracic diameter. The maximum L-L diameter was measured externally at the level of the distal third of the sternum, with the patient standing and the arms abducted, using a dedicated rigid centimeter-calibrated ruler positioned transversely across the chest. The minimum A-P diameter was measured noninvasively from the parasternal long-axis echocardiographic view and defined as the internal distance between the posterior surface of the sternum, corresponding to the true apex of the ultrasound sector, and the anterior surface of the vertebral body. The descending thoracic aorta, visualized posterior to the LA, served as an anatomical landmark for identifying the anterior vertebral surface and standardizing measurement orientation. Both thoracic diameters were measured at end-inspiration and expressed in centimeters. The MHI was subsequently calculated as the ratio of the maximum L-L to the minimum A-P thoracic diameter. This methodology enables quantitative characterization of chest wall conformation without additional radiological imaging or ionizing radiation exposure and was originally developed and validated against conventional radiological assessment [27]. In particular, echocardiographic measurement of the A-P diameter provides a direct estimate of the space available to the heart within the A-P dimension of the thoracic cage. In the present study, particular emphasis was placed on A-P thoracic diameter as a continuous variable, given the specific objective of investigating its relationship with exercise-induced electrocardiographic abnormalities.

Mitral valve morphology was carefully evaluated from standard echocardiographic views. MVP was defined as systolic displacement of one or both mitral leaflets > 2 mm above the mitral annular plane in the parasternal long-axis view [34,35]. The presence and severity of associated MR were assessed using an integrated echocardiographic approach [36]. Mitral annular disjunction (MAD), when present, was also identified and quantified from the parasternal long-axis view as part of the comprehensive assessment of the mitral valve apparatus [37].

Following resting TTE, carotid ultrasonography was performed by the same operator using the same Philips Sparq ultrasound platform equipped with a 12-MHz linear-array transducer. Both carotid arteries were systematically examined for subclinical atherosclerotic involvement. Maximum carotid intima-media thickness (IMT) was measured, and carotid plaque was defined as a focal IMT >1.5 mm [38]. These measurements provided an additional non-coronary marker of atherosclerotic burden to complement the clinical cardiovascular risk profile and CCTA findings.

### 2.3. Exercise Stress Electrocardiography and Echocardiography

All participants underwent symptom-limited ESE using a semi-supine bicycle ergometer (Ergoselect 1200 ELP; Ergoline, Bitz, Germany). Exercise was initiated at a workload of 25 W and progressively increased by 25 W every 2 min to the maximum individually tolerated workload, according to the prespecified protocol [39]. Throughout the examination, patients were continuously monitored for symptoms, hemodynamic response, cardiac rhythm, exercise-induced arrhythmias, and electrocardiographic evidence of myocardial ischemia. Exercise was terminated when patients were unable or unwilling to continue because of fatigue or symptoms, or when clinically appropriate test-termination criteria were reached.

A standard 12-lead ECG was continuously monitored throughout exercise and recovery. Heart rate (HR), systolic blood pressure (SBP), diastolic blood pressure (DBP), and peripheral oxygen saturation were recorded at baseline, during each exercise stage, at peak exercise, and during recovery. Exercise-induced hypertension was defined as a peak exercise SBP ≥ 210 mmHg in men or ≥190 mmHg in women [40]. Maximum workload was expressed in watts, and exercise capacity was additionally expressed as metabolic equivalents (METs). The percentage of age-predicted maximal HR achieved was calculated to characterize the chronotropic response to exercise. The rate-pressure product was calculated from peak HR and peak SBP as an estimate of myocardial workload. Exercise-related symptoms, including dyspnea, chest discomfort, and palpitations, as well as the occurrence and complexity of exercise-induced arrhythmias, were systematically recorded.

Exercise-induced ST-segment changes were evaluated throughout the test and recovery period. An exercise ECG was considered positive for ischemia when exercise induced horizontal or downsloping ST-segment depression ≥ 1.0 mm (0.10 mV), measured 60 ms after the J-point, or upsloping ST-segment depression ≥ 1.5 mm (0.15 mV), measured 80 ms after the J-point, relative to the resting ST level [41]. ST-segment morphology was categorized as pure upsloping, horizontal, downsloping, or mixed/non-pure upsloping. Importantly, ST-segment morphology was classified in all participants according to the configuration observed during exercise, irrespective of whether the magnitude of ST-segment depression fulfilled the prespecified diagnostic criteria for a positive exercise ECG. Thus, the presence of an upsloping, horizontal, downsloping, or mixed ST-segment morphology did not by itself define ECG positivity. The magnitude of exercise-induced ST-segment depression was quantified in the leads exhibiting the greatest dynamic change, with particular attention to inferolateral leads. This continuous assessment allowed characterization of both the presence and magnitude of exercise-induced repolarization abnormalities.

All echocardiographic examinations were performed by the same experienced cardiologist, with patients maintained on the semi-supine bicycle ergometer throughout exercise. Two-dimensional and Doppler echocardiographic images were acquired at rest, during exercise, and at peak exercise. The protocol included serial assessment of LV systolic function, transmitral E/A ratio, average E/e′ ratio, MR severity, and systolic pulmonary artery pressure. Changes from rest to peak exercise (Δ) were calculated for the principal hemodynamic and echocardiographic variables to characterize cardiovascular reserve and dynamic exercise response.

Particular attention was paid to LV regional wall motion throughout the examination. Regional contractile function was assessed by evaluating systolic myocardial thickening and endocardial excursion according to the standard 16-segment LV model. An ESE was considered negative for inducible myocardial ischemia when all adequately visualized LV segments exhibited a normal or hypercontractile response to exercise, without development of new or worsening regional wall-motion abnormalities, irrespective of the concomitant exercise ECG response [39]. Conversely, a positive ESE was defined by the development of new or worsening regional wall-motion abnormalities, corresponding to an increase in wall-motion score by ≥1 grade in at least two adjacent LV segments [39].

Accordingly, electrocardiographic and echocardiographic responses were interpreted as complementary but distinct components of the same exercise examination. For the purposes of the present analysis, particular attention was directed toward patients developing significant exercise-induced ST-segment depression despite a completely normal or hypercontractile LV wall-motion response (ECG+/ESE−), thereby allowing characterization of the discordant phenotype in the absence of echocardiographic evidence of inducible myocardial ischemia.

### 2.4. Study Endpoint

The primary endpoint of the present analysis was the occurrence of a positive exercise ECG despite a negative ESE for inducible myocardial ischemia (ECG+/ESE−). This discordant phenotype was defined as the development of significant exercise-induced ST-segment depression according to the prespecified electrocardiographic criteria, in the presence of a normal or hypercontractile LV wall-motion response without new or worsening regional wall-motion abnormalities during exercise.

Accordingly, the primary objective was to identify clinical, anthropometric, hemodynamic, electrocardiographic, and echocardiographic factors independently associated with the ECG+/ESE− phenotype among symptomatic patients undergoing evaluation for suspected obstructive CAD. Particular attention was directed toward thoracic geometry, and specifically A-P thoracic diameter, as a potential extracardiac determinant of exercise-induced repolarization abnormalities in the absence of stress-induced LV wall-motion abnormalities.

A secondary objective was to evaluate the discriminatory performance of A-P thoracic diameter for identifying patients exhibiting an ECG+/ESE− response. Receiver-operating-characteristic (ROC) analysis was therefore used to quantify its discriminatory ability and to explore the optimal A-P thoracic diameter threshold associated with exercise ECG positivity.

Additional exploratory analyses compared the clinical, anthropometric, hemodynamic, electrocardiographic, and echocardiographic profiles of patients with ECG+/ESE− and ECG−/ESE− responses. In addition, the relationships between A-P thoracic diameter and selected continuous measures of exercise response—including exercise capacity, percentage of age-predicted maximal heart rate achieved, magnitude of exercise-induced ST-segment depression, and peak-exercise E/e′ ratio—were explored across the overall cohort to further characterize the physiological profile associated with different thoracic configurations.

### 2.5. Statistical Analysis

No dedicated sample-size calculation was performed for the present analysis, which was conducted as an exploratory analysis within the prospective MVP&ESE project. All consecutive participants fulfilling the study-specific eligibility criteria and having complete ESE and CCTA data were included.

Continuous variables were assessed for normality using the Shapiro–Wilk test and are presented as mean ± standard deviation for normally distributed variables or median [interquartile range] for non-normally distributed variables, as appropriate. Categorical variables are expressed as absolute numbers and percentages. For comparative analyses, the study population was stratified according to exercise ECG response into patients with a positive exercise ECG despite negative ESE (ECG+/ESE−) and those with both a negative exercise ECG and negative ESE (ECG−/ESE−). Baseline demographic, clinical, anthropometric, laboratory, hemodynamic, electrocardiographic, and echocardiographic characteristics, as well as exercise-test findings, were compared between the two groups. Normally distributed continuous variables were compared using the independent-samples Student’s *t*-test, whereas non-normally distributed variables were compared using the Mann–Whitney U test. Categorical variables were compared using the chi-square test or Fisher’s exact test, as appropriate.

Univariable logistic regression analysis was performed to identify clinical, anthropometric, hemodynamic, electrocardiographic, and echocardiographic factors associated with a positive exercise ECG despite negative ESE. Odds ratios (ORs) with corresponding 95% confidence intervals (CIs) were calculated for each candidate predictor. Variables considered clinically relevant and/or showing an association at univariable analysis were evaluated for inclusion in a parsimonious multivariable logistic regression model. Given the limited number of ECG+/ESE− events, the number of covariates was deliberately restricted to minimize the risk of model overfitting, and redundant or strongly collinear variables were not entered simultaneously. The multivariable model included exercise-induced hypertension, MVP, and A-P thoracic diameter, which were the variables significantly associated with exercise ECG positivity at univariable analysis. Estimated PTP of obstructive CAD was evaluated at univariable analysis but was not entered into the multivariable model because it was not significantly associated with the outcome. A-P thoracic diameter was modeled as a continuous variable, with the OR expressed per 1-cm increase.

Given the limited number of ECG+/ESE− events and the possibility of sparse-data bias or quasi-complete separation, the robustness of the multivariable findings was additionally assessed using Firth penalized logistic regression, including the same three predictors as the primary multivariable model. To assess whether the association between A-P thoracic diameter and exercise ECG positivity was influenced by overall body size, an additional Firth penalized sensitivity model was fitted including BSA together with exercise-induced hypertension, MVP, and A-P thoracic diameter. Using the same penalized approach, the relationship between A-P thoracic diameter and exercise ECG positivity was examined separately in patients with and without MVP. Potential effect modification by MVP status was formally assessed in the overall cohort by including continuous A-P thoracic diameter, MVP status, and their interaction term (A-P thoracic diameter × MVP) in a Firth penalized model. ORs with corresponding 95% CIs and p values were reported for the penalized analyses.

ROC curve analysis was performed to assess the ability of A-P thoracic diameter to discriminate between patients with and without an ECG+/ESE− response. Discriminatory performance was quantified by the area under the ROC curve (AUC) with its corresponding 95% CI. The optimal A-P thoracic diameter threshold was identified using the maximum Youden index, and the corresponding sensitivity and specificity were calculated. Internal validation of the discriminatory performance of A-P thoracic diameter was performed using 5000 bootstrap resamples. Optimism was estimated as the mean difference between the AUC obtained in each bootstrap sample and the AUC obtained when the corresponding bootstrap-derived model was applied to the original dataset. The optimism-corrected AUC was calculated by subtracting the estimated optimism from the apparent AUC.

Pearson’s correlation analysis was used to examine the relationships between A-P thoracic diameter and selected continuous measures of exercise response, including achieved METs, percentage of age-predicted maximal heart rate achieved, magnitude of exercise-induced ST-segment depression, and peak-exercise E/e′ ratio. Pearson correlation coefficients (r) and corresponding p values were reported. Given the magnitude of the observed correlations, robustness was additionally assessed using Spearman rank correlation coefficients (ρ). Distributional assumptions of the variables included in the correlation analyses were evaluated using the Shapiro–Wilk test. Sensitivity analyses were also performed after exclusion of potentially influential observations, defined as observations with Cook’s distance > 4/n and/or an absolute externally studentized residual > 3, and Pearson correlation coefficients were recalculated in the resulting datasets.

Reproducibility of A-P thoracic diameter measurement was assessed in a subgroup of 19 patients using a two-way random-effects, absolute-agreement, single-measure intraclass correlation coefficient [ICC(2,1)]. Intra-observer reproducibility was evaluated by comparing the initial A-P thoracic diameter measurement with a repeat measurement performed by the same operator, whereas inter-observer reproducibility was assessed by comparing the initial measurement with an independent measurement performed by a second operator. Agreement was additionally evaluated using Bland–Altman analysis, with mean differences and 95% limits of agreement reported. The 95% CIs for ICC estimates were obtained using bootstrap resampling.

All statistical tests were two-sided, and a *p* value < 0.05 was considered statistically significant. Given the exploratory nature of the analysis and the relatively small sample size, no adjustment for multiple comparisons was performed.

Statistical analyses were performed using IBM SPSS Statistics, version 28 (IBM Corp., Armonk, NY, USA).

### 2.6. Use of Artificial Intelligence Tools

During the preparation of this manuscript, ChatGPT (OpenAI, San Francisco, CA, USA; GPT-5.6 Sol) was used as an AI-assisted tool exclusively for language and editorial support and for graphical assistance. Specifically, the tool was used to improve English language, grammar, sentence structure, stylistic consistency, and overall readability, as well as to support the graphical design and visual refinement of the manuscript figures. The AI tool was not involved in the study conception or design, patient recruitment, data collection, data processing, statistical analysis, interpretation of the results, or formulation of the scientific conclusions. All methodological decisions, scientific interpretations, and intellectual content were developed and critically evaluated by the authors. All AI-assisted text and graphical material were subsequently reviewed, revised where necessary, and approved by the authors to ensure scientific accuracy and faithful representation of the intended message. The authors retain full responsibility for the accuracy, integrity, originality, and scientific content of the final manuscript.

## 3. Results

### 3.1. Baseline Characteristics of the Study Population

A total of 101 consecutive symptomatic individuals prospectively enrolled at our center fulfilled the study-specific eligibility criteria and were included in the present analysis. Of these, 35 patients (34.7%) exhibited an ECG+/ESE− response, whereas the remaining 66 (65.3%) had a negative exercise ECG and negative ESE (ECG−/ESE−).

Baseline demographic, anthropometric, clinical, laboratory, treatment, and vascular imaging characteristics of the overall population and according to exercise ECG response are summarized in Table 1.

The overall population was predominantly female and had a relatively lean anthropometric profile. Demographic characteristics and conventional cardiovascular risk factors were generally comparable between the ECG+/ESE− and ECG−/ESE− groups. In contrast, thoracic geometry differed significantly: patients with ECG+/ESE− exhibited a smaller A-P thoracic diameter and a higher MHI, whereas L-L thoracic diameter was virtually identical between groups. A history of a previous exercise stress test considered equivocal for myocardial ischemia was significantly more common among patients with ECG+/ESE−.

Importantly, coronary and extracoronary atherosclerotic burden did not appear to account for these differences. CCTA demonstrated no obstructive epicardial CAD in any participant, and the proportions of patients with completely normal coronary arteries or mild non-obstructive atherosclerosis/calcification were nearly identical between groups. Carotid atherosclerotic burden and laboratory parameters were likewise comparable. Ongoing medical therapy was broadly similar between groups, although antiarrhythmic drug use was confined to the ECG−/ESE− group.

### 3.2. Resting Hemodynamic, Electrocardiographic, and Echocardiographic Findings

Resting hemodynamic, electrocardiographic, and echocardiographic findings for the overall population and according to exercise ECG response are detailed in Table 2.

Overall, resting cardiovascular function was preserved, with predominantly normal LV geometry, preserved LV systolic function, low estimated LV filling pressures, and normal right ventricular longitudinal systolic function and pulmonary pressures. Resting blood pressure and heart rate were comparable between the ECG+/ESE− and ECG−/ESE− groups, as was the distribution of intraventricular conduction abnormalities, which predominantly consisted of incomplete right bundle branch block, either isolated or associated with left anterior fascicular block.

Compared with ECG−/ESE− patients, those with ECG+/ESE− showed a less pronounced LV structural profile, characterized by lower posterior wall thickness and LV mass index, together with a markedly smaller LA A-P diameter. Resting LVEF and E/e′ were similar between groups, whereas the E/A ratio was slightly higher in the ECG+/ESE− group. MVP was significantly more frequent among patients with ECG+/ESE−, while the prevalence of MAD and the distribution of MR severity were comparable between groups. Notably, among patients with MAD, disjunction distance was significantly smaller in the ECG+/ESE− group.

Right-heart parameters were generally similar between groups, although TAPSE was modestly higher in ECG+/ESE− patients; importantly, values remained within the preserved range in both groups, and resting sPAP was virtually identical.

### 3.3. Exercise Stress Echocardiography Findings

All patients completed symptom-limited ESE with adequate diagnostic image quality. Detailed exercise capacity, hemodynamic, electrocardiographic, arrhythmic, symptomatic, and echocardiographic findings for the overall population and according to exercise ECG response are reported in Table 3.

Overall exercise capacity was moderate. Maximum workload and achieved METs were significantly lower in the ECG+/ESE− group than in the ECG−/ESE− group, indicating reduced exercise tolerance in patients with exercise ECG positivity. Patients with ECG+/ESE− achieved a higher peak heart rate and showed a greater increase in heart rate from rest to peak exercise. Peak systolic blood pressure and the increase in systolic blood pressure from rest to peak exercise were numerically higher in the ECG+/ESE− group, but these differences were not statistically significant. Diastolic blood pressure responses were also comparable between groups. Nevertheless, exercise-induced hypertension was significantly more frequent among ECG+/ESE− patients, suggesting that an exaggerated systolic blood pressure response occurred more frequently in this phenotype despite the absence of significant differences in mean peak SBP or ΔSBP. Peripheral oxygen saturation remained stable and comparable between groups throughout exercise.

Despite uniformly negative stress echocardiographic findings, with a normal or hypercontractile LV response and no new or worsening regional wall-motion abnormalities, 35 patients (34.7%) developed a positive exercise ECG according to the prespecified criteria. As expected from the definition of the two groups, the magnitude of exercise-induced inferolateral ST-segment depression was greater in ECG+/ESE− patients. Although ST-segment morphology was categorized in all participants irrespective of diagnostic ECG positivity, its distribution did not differ significantly between groups. Importantly, none of the ECG−/ESE− patients reached the prespecified magnitude criteria for a positive exercise ECG. Exercise-related arrhythmic burden and symptom occurrence also did not differ significantly between groups.

Peak-exercise echocardiography demonstrated preserved LV contractile reserve in both groups. Peak LVEF, E/e′, and sPAP, as well as their changes from rest to peak exercise, were comparable between ECG+/ESE− and ECG−/ESE− patients. MR remained non-severe throughout exercise, with no significant between-group differences in peak MR severity. Thus, exercise ECG positivity occurred in the context of reduced exercise tolerance and a more pronounced chronotropic response, with a higher prevalence of exercise-induced hypertension but no significant difference in mean peak or exercise-induced change in systolic blood pressure, and without corresponding evidence of greater exercise-induced LV filling-pressure elevation, impaired contractile reserve, or pulmonary pressure response.

### 3.4. Thoracic Geometry and Its Relationship with Exercise Response

**Figure 1:**
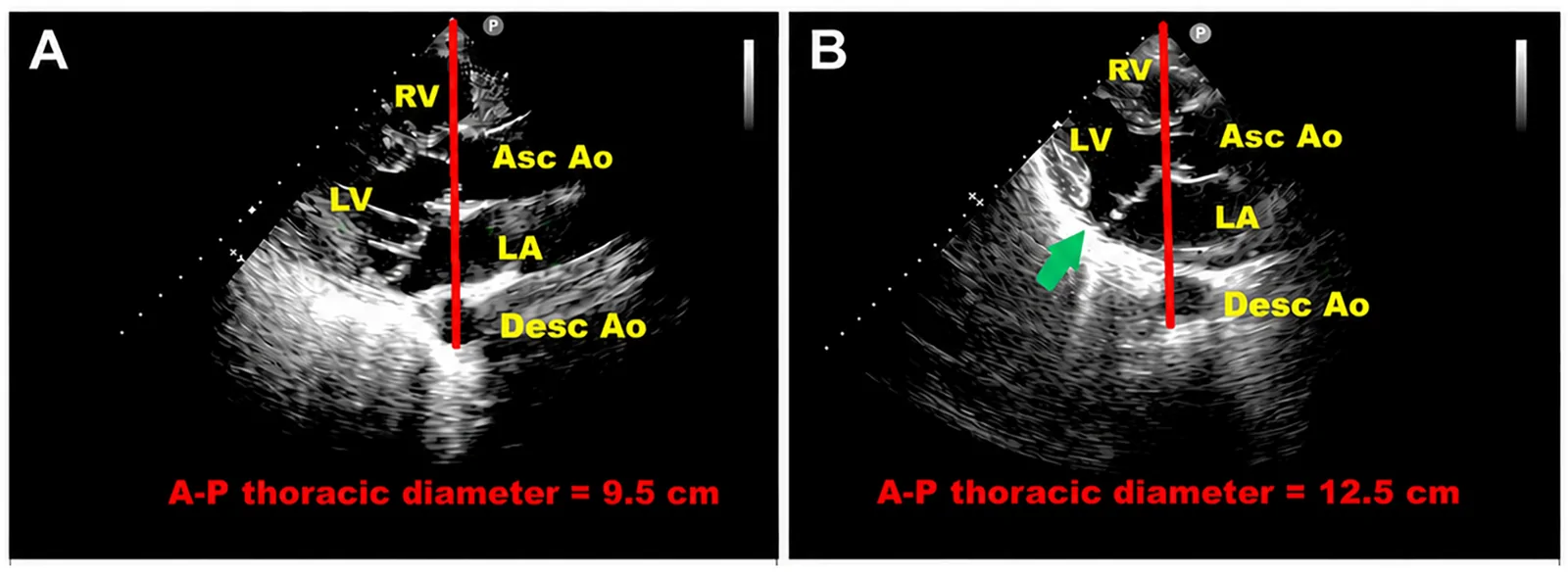
Representative parasternal long-axis echocardiographic views illustrating two patients with a positive exercise ECG despite negative exercise stress echocardiography (ECG+/ESE−), both presenting with narrow chest wall conformation and mitral valve prolapse (MVP). A-P thoracic diameters were 9.5 cm (**A**) and 12.5 cm (**B**), respectively. In both panels, the red line indicates the A-P thoracic diameter, measured at end-inspiration as the distance between the posterior surface of the sternum and the anterior surface of the vertebral body, using the descending thoracic aorta as an anatomical landmark. In panel (**B**), the green arrow indicates associated mitral annular disjunction (MAD). A-P, antero-posterior; Asc Ao, ascending aorta; Desc Ao, descending aorta; ECG, electrocardiogram; ESE, exercise stress echocardiography; LA, left atrium; LV, left ventricle; MAD, mitral annular disjunction; MVP, mitral valve prolapse; RV, right ventricle.

Representative parasternal long-axis echocardiographic examples of thoracic A-P diameter assessment in two patients with the ECG+/ESE− phenotype and concomitant MVP are shown in Figure 1. Both patients exhibited a narrow chest wall configuration, with A-P thoracic diameters of 9.5 and 12.5 cm, respectively. These representative cases also illustrate the close anatomical interplay between thoracic geometry and the mitral valve apparatus, with associated MAD visible in one patient.

**Figure 2:**
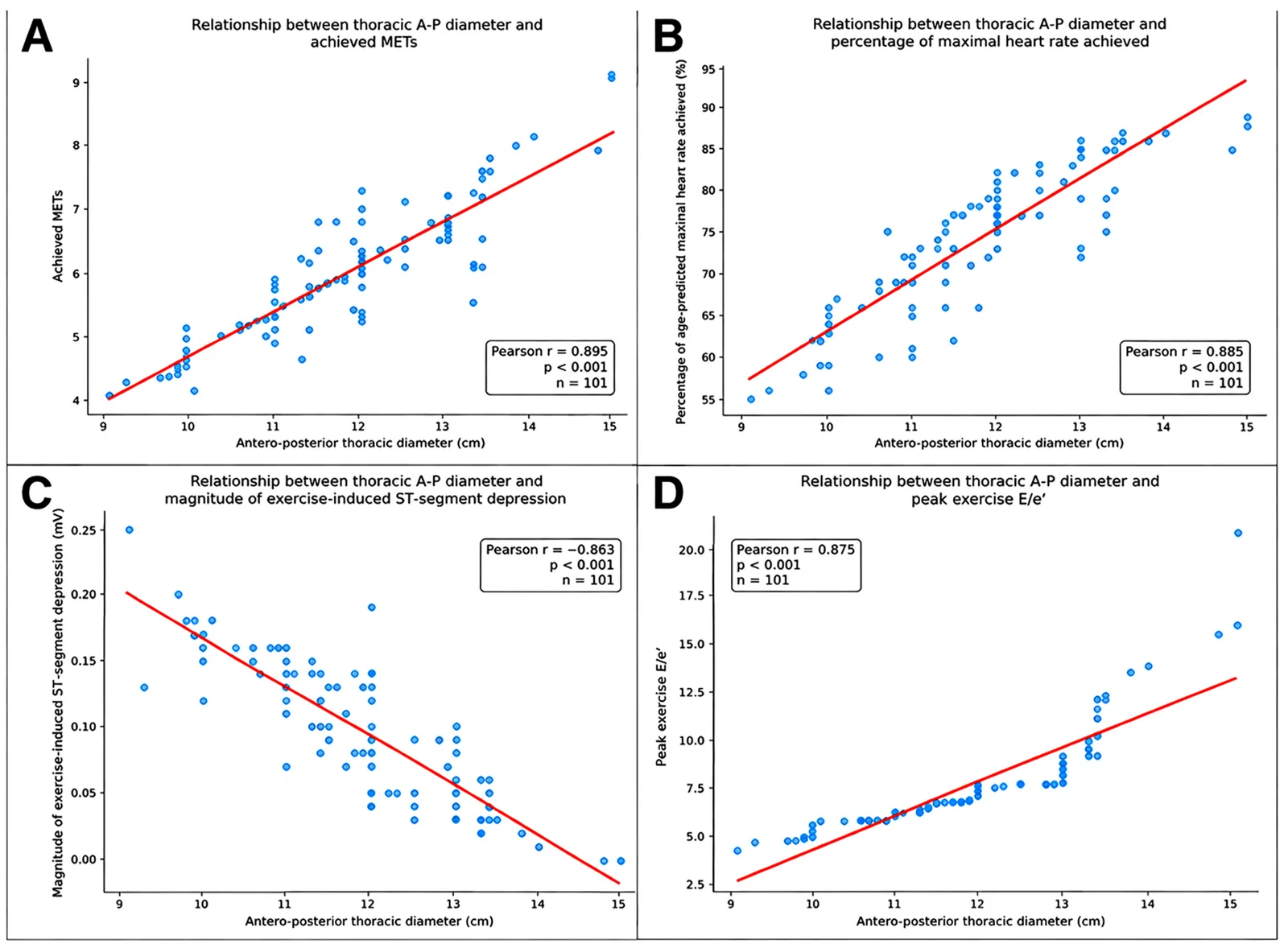
Relationships between antero-posterior thoracic diameter and exercise-test findings. Scatterplots illustrate the relationships between A-P thoracic diameter and achieved metabolic equivalents (**A**), percentage of age-predicted maximal heart rate achieved (**B**), magnitude of exercise-induced ST-segment depression (**C**), and peak-exercise E/e′ ratio (**D**). A-P thoracic diameter was strongly and directly correlated with exercise capacity, percentage of age-predicted maximal heart rate achieved, and peak-exercise E/e′ ratio, and strongly and inversely correlated with the magnitude of exercise-induced ST-segment depression (all *p* < 0.001). Pearson correlation coefficients and sample size are reported within each panel. A-P, antero-posterior; METs, metabolic equivalents.

A strong relationship emerged between thoracic geometry and multiple components of the exercise response (Figure 2).

A-P thoracic diameter showed a strong positive correlation with achieved exercise capacity (METs; Pearson r = 0.895, *p* < 0.001) and with the percentage of age-predicted maximal heart rate achieved (r = 0.885, *p* < 0.001). Conversely, A-P thoracic diameter was strongly and inversely correlated with the magnitude of exercise-induced ST-segment depression (r = −0.863, *p* < 0.001), indicating progressively greater repolarization abnormalities with decreasing A-P thoracic diameter. Peak-exercise E/e′ was also strongly and positively correlated with A-P thoracic diameter (r = 0.875, *p* < 0.001). Rank-based analyses confirmed the direction and magnitude of all four associations (Spearman ρ = 0.902 for METs, 0.881 for percentage of age-predicted maximal heart rate, −0.881 for ST-segment depression, and 0.996 for peak-exercise E/e′; all *p* < 0.001). Sensitivity analyses excluding potentially influential observations similarly showed persistently strong Pearson correlations (r = 0.914, 0.908, −0.914, and 0.914, respectively; all *p* < 0.001), indicating that the observed relationships were not driven by isolated influential data points. Thus, smaller A-P thoracic dimensions were consistently associated with lower exercise capacity, a blunted chronotropic response, greater exercise-induced ST-segment depression, and lower peak-exercise E/e′ values.

### 3.5. Factors Associated with a Positive Exercise ECG

Univariable and multivariable logistic regression analyses were performed to identify factors associated with a positive exercise ECG despite negative ESE. The complete regression results are summarized in Table 4.

At univariable analysis, exercise-induced hypertension and MVP were associated with higher odds of exercise ECG positivity, whereas increasing A-P thoracic diameter showed a strong inverse association. Estimated PTP of obstructive CAD was not significantly associated with the ECG+/ESE− phenotype.

In the parsimonious multivariable model, MVP and A-P thoracic diameter remained independently associated with exercise ECG positivity, whereas exercise-induced hypertension no longer reached statistical significance after adjustment. Specifically, MVP was associated with higher odds of exercise ECG positivity (adjusted OR 8.50, 95% CI 1.07–67.75; *p* = 0.043), whereas increasing A-P thoracic diameter showed a strong inverse association (adjusted OR per 1-cm increase 0.026, 95% CI 0.005–0.143; *p* < 0.001). Exercise-induced hypertension was no longer statistically significant after adjustment (adjusted OR 5.74, 95% CI 0.64–51.52; *p* = 0.119). The wide confidence intervals surrounding some multivariable estimates, particularly those for MVP and exercise-induced hypertension, indicate limited precision and should be considered when interpreting the magnitude of these associations.

In the Firth penalized model, A-P thoracic diameter remained strongly and inversely associated with exercise ECG positivity (OR per 1-cm increase 0.045, 95% CI 0.011–0.177; *p* < 0.001), whereas MVP showed a borderline positive association (OR 6.24, 95% CI 0.99–39.39; *p* = 0.051) and exercise-induced hypertension was not statistically significant (OR 4.20, 95% CI 0.59–29.87; *p* = 0.151). After additional adjustment for BSA using Firth penalized regression, the inverse association with A-P thoracic diameter remained significant (OR 0.0055, 95% CI 0.0002–0.149; *p* = 0.002), while MVP was also independently associated with ECG positivity (OR 22.97, 95% CI 1.92–275.19; *p* = 0.013). In analyses stratified by MVP status, A-P thoracic diameter remained inversely associated with ECG positivity both in patients without MVP (OR 0.178, 95% CI 0.061–0.519; *p* = 0.002) and in those with MVP (OR 0.00138, 95% CI 0.000010–0.185; *p* = 0.008), although the latter estimate was highly imprecise. A significant A-P thoracic diameter × MVP interaction was observed (*p* = 0.038), suggesting that the magnitude of the association between thoracic geometry and exercise ECG positivity may differ according to MVP status. Given the small subgroup sizes and wide confidence intervals, this interaction should be regarded as exploratory.

### 3.6. Discriminatory Performance of A-P Thoracic Diameter

**Figure 3:**
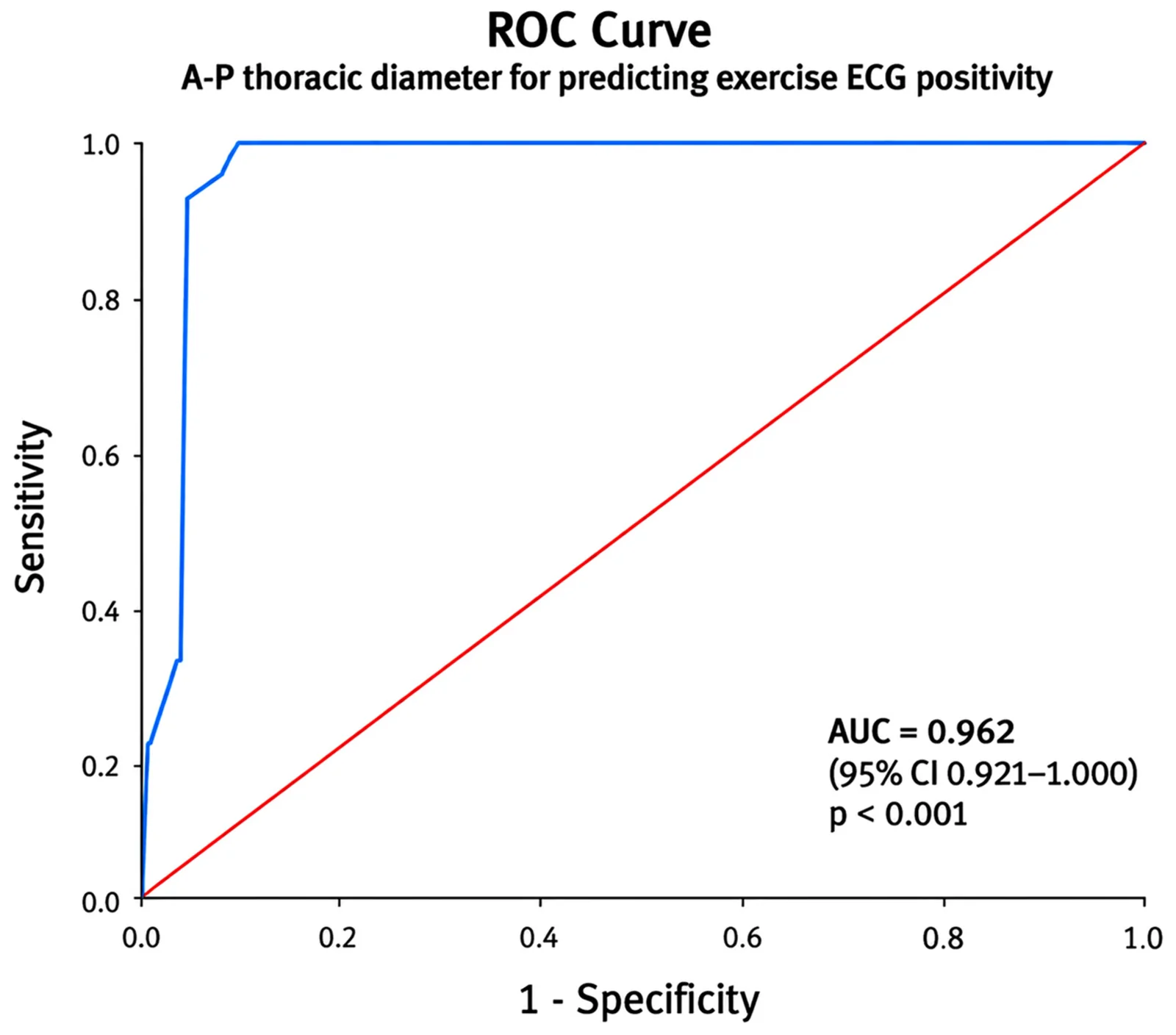
Receiver-operating-characteristic (ROC) curve illustrating the discriminatory performance of A-P thoracic diameter for identifying patients with a positive exercise ECG despite negative exercise stress echocardiography (ECG+/ESE−). The area under the curve (AUC) was 0.962 (95% CI, 0.921–1.000; *p* < 0.001). The optimal data-derived threshold, identified using the maximum Youden index, was ≤10.95 cm, yielding 97.1% sensitivity and 93.9% specificity. The blue line represents the ROC curve, and the red diagonal line represents the reference line corresponding to no discriminatory ability (AUC = 0.50). A-P, antero-posterior; AUC, area under the curve; CI, confidence interval; ECG, electrocardiography; ESE, exercise stress echocardiography; ROC, receiver-operating-characteristic.

ROC analysis demonstrated excellent discriminatory performance of A-P thoracic diameter for identifying patients with a positive exercise ECG despite negative ESE. The area under the ROC curve was 0.962 (standard error, 0.021; 95% CI, 0.921–1.000; *p* < 0.001), indicating excellent separation according to exercise ECG response (Figure 3).

Internal validation using 5000 bootstrap resamples showed negligible optimism in discrimination (mean optimism < 0.001), yielding an optimism-corrected AUC of 0.962.

Based on the maximum Youden index, an A-P thoracic diameter ≤ 10.95 cm represented the optimal data-derived threshold for identifying exercise ECG positivity, yielding a sensitivity of 97.1% and a specificity of 93.9% (Youden index, 0.910). This high discriminatory performance was consistent with the continuous inverse association observed in logistic regression, whereby progressively larger A-P thoracic diameters were associated with a lower likelihood of the ECG+/ESE− phenotype.

### 3.7. Reproducibility of A-P Thoracic Diameter Measurement

Reproducibility of A-P thoracic diameter measurement was assessed in a subgroup of 19 patients. Intra-observer reproducibility was high, with an ICC(2,1) of 0.961 (95% CI, 0.920–0.979). Bland–Altman analysis showed a mean difference of 0.16 cm, with 95% limits of agreement from −0.42 to 0.74 cm. Inter-observer reproducibility was also high, with an ICC(2,1) of 0.901 (95% CI, 0.805–0.943), a mean difference of 0.28 cm, and 95% limits of agreement from −0.64 to 1.20 cm. Overall, these findings indicate high intra- and inter-observer reproducibility of A-P thoracic diameter measurement in the examined subgroup.

## 4. Discussion

### 4.1. Principal Findings

The present prospective study provides new insights into the determinants of a positive exercise ECG despite negative ESE and absence of obstructive CAD in symptomatic patients. Exercise-induced ST-segment depression occurred in approximately one-third of the cohort, confirming that electrocardiographic and mechanical responses to exercise may diverge substantially. Importantly, ECG+/ESE− patients exhibited a distinctive thoraco-cardiac phenotype characterized by narrower A-P thoracic geometry, more frequent MVP, and reduced exercise capacity, despite preserved LV contractile reserve and without evidence of greater exercise-induced filling-pressure or pulmonary pressure responses.

Among the clinical, anthropometric, hemodynamic, and echocardiographic variables investigated, MVP and A-P thoracic diameter were independently associated with exercise ECG positivity in the primary multivariable model, although A-P thoracic diameter emerged as the more consistent finding across complementary analyses. In Firth penalized regression, the inverse association between A-P thoracic diameter and ECG positivity remained robust, whereas the association with MVP was of borderline statistical significance. Importantly, the association with A-P thoracic diameter persisted after additional adjustment for BSA and was also observed separately in patients with and without MVP, indicating that its relationship with exercise ECG positivity was not confined to the MVP phenotype. At the same time, the significant A-P thoracic diameter × MVP interaction suggested that the magnitude of this association may differ according to MVP status, with an apparently stronger association among patients with MVP. Given the limited subgroup sizes and imprecision of the corresponding effect estimates, however, this potential effect modification should be regarded as exploratory rather than confirmatory. Taken together, the findings suggest that restricted A-P thoracic geometry may represent an anatomical correlate of exercise-induced repolarization abnormalities that is not restricted to the MVP phenotype, while MVP may identify a thoraco-cardiac context in which this relationship is accentuated.

The association with A-P thoracic diameter extended beyond the dichotomous ECG classification: progressively smaller A-P dimensions were associated with lower exercise capacity and chronotropic response, greater ST-segment depression, and lower peak-exercise E/e′. These convergent relationships suggest that thoracic geometry may capture a broader exercise-response phenotype rather than simply represent an anatomical correlate of ECG positivity.

Collectively, these findings support restricted thoracic geometry as a potential extracardiac contributor to ECG–ESE discordance. The coexistence of a narrow A-P chest configuration, frequent MVP, exercise-induced repolarization abnormalities, and preserved stress-induced LV contractile reserve raises the hypothesis of a thoraco-cardiac mechanical phenotype in which altered cardiac geometry and mechanical interactions may influence repolarization during exercise. This interpretation does not exclude coronary microvascular or vasomotor mechanisms, which were not directly assessed, nor does it suggest that thoracic geometry should replace conventional CAD evaluation. Rather, thoracic geometry may provide complementary phenotypic information when exercise ECG and stress echocardiographic findings are discordant.

### 4.2. Comparison with Previous Studies

Discordant ECG+/ESE− findings have been repeatedly described, although their clinical and prognostic significance remains controversial. In 1725 patients with chest pain and no previous CAD, Hwang et al. [12] observed ECG+/Echo− findings in 11.1% of the study population, without a significant increase in adjusted cardiovascular risk. Similarly, Chai et al. [42] reported ECG+/Echo− discordance in 122 of 793 patients with negative stress echocardiography, without evidence of increased cardiovascular events during an 18-month follow-up. These findings are consistent with the traditionally reassuring prognostic value attributed to a normal stress-induced LV wall-motion response [43,44,45].

Conversely, larger studies have suggested that discordant ECG+/ESE− findings should not invariably be considered benign. In the Duke cohort of 15,077 patients, Daubert et al. [13] identified ECG+/Echo− discordance in 8.5% of participants. This phenotype was associated with more frequent downstream non-invasive testing and coronary angiography and with higher short- and long-term event rates. Older age, hyperlipidemia, higher resting systolic blood pressure, and lower resting heart rate were independently associated with ECG+/Echo− findings. Over a median follow-up of 7.3 years, the composite event rate was 14.6% among ECG+/Echo− patients compared with 8.5% among ECG−/Echo− patients, although it remained substantially lower than that observed in patients with positive stress echocardiography. A subsequent meta-analysis of nine studies including 23,715 patients similarly reported a 33% higher risk of death or myocardial infarction among patients with discordant stress-test findings [46].

More recently, Duran et al. [47] demonstrated a greater burden of coronary calcification among individuals with abnormal exercise ECG but normal stress echocardiography, who were approximately 4.5-fold more likely to have a CAC score ≥ 400 after multivariable adjustment. These findings suggest that, at least in some patients, an abnormal exercise ECG may identify subclinical coronary atherosclerotic burden even when stress-induced regional wall motion remains normal. However, that population was predominantly asymptomatic, and the analysis focused on coronary calcium rather than obstructive epicardial disease.

Of note, the present cohort illustrates a potential dissociation between conventional cardiovascular risk-factor burden and the anatomical substrate underlying exercise-induced ECG abnormalities. Hypertension and dyslipidemia were relatively common, affecting 58.4% and 67.3% of participants, respectively, yet the median estimated PTP of obstructive CAD was only 9%. Moreover, approximately half of the study population had completely normal coronary arteries on CCTA, while the remainder had only mild non-obstructive atherosclerosis or calcification and no participant had obstructive CAD. Extracoronary atherosclerotic burden was also relatively limited, with carotid plaques detected in 24.8% of patients. Thus, although conventional cardiovascular risk factors remained prevalent, they alone appear insufficient to explain the high occurrence of exercise-induced ST-segment abnormalities in this selected population. This apparent dissociation further supports the possibility that non-obstructive coronary mechanisms and non-coronary determinants, including hemodynamic, autonomic, structural, and thoraco-cardiac mechanical factors, may contribute to ECG+/ESE− discordance.

The present study addresses a complementary clinical scenario. All participants were symptomatic, all had negative ESE, and CCTA demonstrated either normal coronary arteries or only mild non-obstructive coronary atherosclerosis/calcification. Therefore, flow-limiting epicardial stenosis was unlikely to explain the observed ST-segment abnormalities. Within this specifically selected population, conventional estimated PTP was not independently associated with exercise ECG positivity, whereas A-P thoracic diameter and MVP were. These findings do not contradict previous evidence linking ECG+/ESE− discordance to coronary atherosclerosis; rather, they underscore the heterogeneity of this phenotype. In some patients, discordance may reflect underlying atherosclerotic or microvascular disease, whereas in others structural, autonomic, hemodynamic, or extracardiac mechanical factors may predominate.

### 4.3. Pathophysiological Mechanisms Underpinning ST-Segment Depression in the Absence of Obstructive CAD

Exercise-induced ST-segment depression in the absence of obstructive CAD represents a heterogeneous phenomenon and should not automatically be interpreted as evidence of flow-limiting epicardial coronary disease. Several clinical, hemodynamic, autonomic, and structural factors have been associated with false-positive or discordant exercise ECG responses, including female sex, low PTP of CAD, hypertensive response to exercise, hyperventilation, MVP, and Takotsubo-like patterns. Previous studies have particularly emphasized the higher prevalence of apparently false-positive exercise ECG findings in women [17,18] and in individuals with a low baseline likelihood of obstructive CAD [19]. An exaggerated blood pressure response to exercise may increase myocardial oxygen demand and transiently alter subendocardial perfusion despite angiographically unobstructed epicardial coronary arteries [20,21,22,23]. Consistent with this mechanism, exercise-induced hypertension showed a strong association with ECG positivity at univariable analysis in the present cohort (OR 6.67), but did not retain statistical significance after multivariable adjustment. This finding suggests that an exaggerated blood pressure response may contribute to exercise-induced ST-segment abnormalities, while its effect may overlap with other clinical, anatomical, or hemodynamic characteristics. Importantly, the extremely wide confidence interval surrounding the adjusted estimate indicates substantial statistical uncertainty and precludes firm conclusions regarding its independent contribution to the ECG+/ESE− phenotype. Similarly, transient catecholamine excess and enhanced adrenergic stimulation may affect myocardial mechanics and repolarization and, in selected circumstances, produce a Takotsubo-like response characterized by exercise-induced repolarization and contractile abnormalities in the absence of obstructive CAD [24]. Hyperventilation represents another recognized mechanism, particularly in patients with MVP, and may influence cardiac position, autonomic tone, myocardial repolarization, and electrolyte balance [25].

These mechanisms are not mutually exclusive and may coexist within the same patient. Importantly, CCTA provides an accurate anatomical assessment of epicardial coronary arteries but does not directly interrogate coronary microvascular function or dynamic vasomotor abnormalities. Therefore, the absence of obstructive CAD in the present population does not establish a purely non-ischemic origin of exercise-induced ST-segment depression. Nevertheless, the uniformly negative ESE findings, preserved LV contractile reserve, and strong relationships observed between thoracic geometry and multiple components of the exercise response suggest that extracoronary mechanisms may contribute substantially to ECG positivity in at least a subset of these patients.

The relationship between A-P thoracic diameter and peak-exercise E/e′ is particularly intriguing. Smaller A-P thoracic dimensions were associated with greater ST-segment depression but lower peak-exercise E/e′ values. This pattern, together with preserved LV contractile reserve, argues against overt exercise-induced elevation of LV filling pressures or intrinsic myocardial dysfunction as the predominant explanation for reduced exercise tolerance. Instead, it raises the possibility that restricted thoracic geometry and altered thoraco-cardiac interactions may contribute to this functional phenotype. Although exercise E/e′ is an indirect estimate of LV filling pressure and these cross-sectional associations cannot establish causality, the findings support a phenotype in which ECG+/ESE− discordance and reduced exercise tolerance coexist with preserved global contractile reserve and relatively low exercise filling-pressure estimates.

### 4.4. Thoracic Geometry, Mitral Valve Prolapse, and a Potential Thoraco-Cardiac Mechanical Phenotype

The present findings support an additional extrinsic mechanical mechanism related to the close interplay between thoracic geometry, cardiac position, and the mitral valve apparatus. MVP is strongly associated with thoracic skeletal abnormalities and narrow or concave chest wall conformation [48,49,50,51,52]. Previous evidence has also described a characteristic cardiovascular phenotype encompassing nonspecific ST-T abnormalities, ventricular ectopy, relatively small cardiac chambers, and preserved ventricular function [53,54]. Accordingly, chest wall morphology may represent more than an incidental anthropometric characteristic and could influence the mechanical environment in which the heart and mitral apparatus operate.

A reduced A-P thoracic diameter decreases the space available to the heart between the sternum and vertebral column. Chronic anterior mechanical constraint may modify cardiac position and rotation and alter the spatial relationships among the LV, mitral annulus, papillary muscles, and surrounding thoracic structures. In predisposed individuals, these geometric changes may influence mitral valve morphology and function [55]. Mechanical forces transmitted through the mitral apparatus may consequently increase traction along the chordae toward the papillary muscles and adjacent inferobasal and inferolateral myocardium. During exercise, increased LV contractility and greater mitral annular excursion could amplify these mechanical interactions, potentially producing regional mechanical heterogeneity and asynchronous myocardial repolarization despite preserved global contractile reserve and absence of obstructive epicardial CAD.

This hypothesis is consistent with the well-described association between MVP and inferior ST-T abnormalities [56,57] and with evidence that abnormal mechanical tugging of the sub-mitral apparatus may preferentially involve the papillary muscles and basal inferolateral myocardium [58]. The present results provide additional physiological support for this interpretation. Decreasing A-P thoracic diameter was strongly associated with progressively greater exercise-induced ST-segment depression, while simultaneously being associated with lower exercise capacity, a lower percentage of age-predicted maximal heart rate achieved, and lower peak-exercise E/e′. Thus, the narrow-chest phenotype was characterized not merely by greater repolarization abnormalities but by a broader constellation of reduced functional capacity, attenuated chronotropic response, and relatively low estimated LV filling pressures during exercise.

MAD may represent a further component of this mechanical continuum rather than invariably indicating an arrhythmogenic substrate. Excessive annular mobility and MAD can increase traction on the papillary muscles and basal inferolateral LV wall and, in selected patients, may promote myocardial fibrosis and ventricular arrhythmias [59,60,61]. However, MAD is morphologically and clinically heterogeneous. Our previous observations identified a potentially more benign MAD phenotype characterized by a narrow A-P thoracic diameter, reduced basal myocardial strain potentially attributable to extrinsic sternal compression, preserved biventricular function, a low burden of complex ventricular arrhythmias, and favorable mid-term outcome [62]. These observations suggest that the clinical significance of MAD may depend, at least partly, on the broader anatomical and mechanical context in which it occurs.

Importantly, the potential relationship between restricted thoracic geometry and exercise-induced repolarization abnormalities may not be confined to individuals with MVP. In a previous proof-of-concept observation, we described a patient with pectus excavatum and a narrow A-P thoracic diameter without evidence of structural heart disease, who developed marked inferolateral ST-segment depression during exercise despite a hyperkinetic LV response, normal myocardial perfusion imaging, and normal coronary arteries on CCTA [63]. These findings suggested that thoracic constraint itself may contribute to exercise-induced “pseudo-ischemic” repolarization abnormalities even outside the typical MVP-associated phenotype.

Accordingly, MVP, with or without MAD, and reduced A-P thoracic diameter may in some individuals represent interconnected components of a broader thoraco-cardiac mechanical phenotype. Within this framework, restricted thoracic geometry may influence cardiac orientation and basal LV mechanics at rest, while exercise may accentuate mitral annular motion, papillary-muscle tethering, and regional mechanical heterogeneity, ultimately facilitating dynamic repolarization abnormalities without necessarily indicating intrinsic myocardial disease, microvascular dysfunction or obstructive epicardial CAD. This mechanistic framework provides a plausible explanation for the independent associations of both MVP and A-P thoracic diameter with exercise ECG positivity observed in the present study and warrants further investigation through dedicated mechanistic studies integrating thoracic morphometry with advanced myocardial deformation imaging, assessment of coronary microvascular function, and exercise hemodynamics.

### 4.5. Implications for Clinical Practice

Our findings may have practical implications for the interpretation of exercise-test results in symptomatic patients undergoing evaluation for suspected CAD. Measurement of A-P thoracic diameter can be readily incorporated into conventional TTE, requiring only a standard parasternal long-axis view and no additional imaging procedure, ionizing radiation exposure, or substantial examination time. Unlike more complex imaging-derived indices, A-P thoracic diameter represents a simple continuous anatomical parameter that can be obtained during routine echocardiographic examination and interpreted together with cardiac morphology, mitral valve characteristics, and the hemodynamic response to exercise.

This approach may be particularly relevant because previous work has shown that symptomatic patients with MVP and concave chest wall conformation may exhibit small cardiac chamber dimensions, frequent exercise-induced ST-segment abnormalities, and a high prevalence of negative ESE, while maintaining a favorable medium-term prognosis [64]. In this setting, chest-shape assessment may provide complementary information for identifying a phenotype characterized by a lower cardiovascular event burden, with the markedly smaller LA A-P diameter observed in ECG+/ESE− patients representing an additional structural feature of this distinctive thoraco-cardiac remodeling pattern. A reduced thoracic A-P dimension restricts the space available to the heart along the same antero-posterior axis and may therefore be accompanied by smaller A-P dimensions of the cardiac chambers, including the LA. In this framework, reduced LA A-P diameter may primarily represent a geometric consequence or marker of restricted thoracic configuration rather than an independent determinant of exercise-induced repolarization abnormalities. However, given the cross-sectional nature of these observations, the direction of this anatomical relationship cannot be definitively established. Thus, thoracic and cardiac geometry may provide complementary information when conventional exercise ECG and stress imaging yield apparently discordant results.

The present findings extend this concept by suggesting that A-P thoracic diameter should be interpreted as part of a broader clinical and echocardiographic phenotype rather than in isolation. Its independent association with exercise ECG positivity, together with its relationships with exercise capacity, chronotropic response, ST-segment depression, and peak-exercise E/e′, supports the potential value of integrating thoracic geometry with conventional exercise and echocardiographic findings.

**Figure 4:**
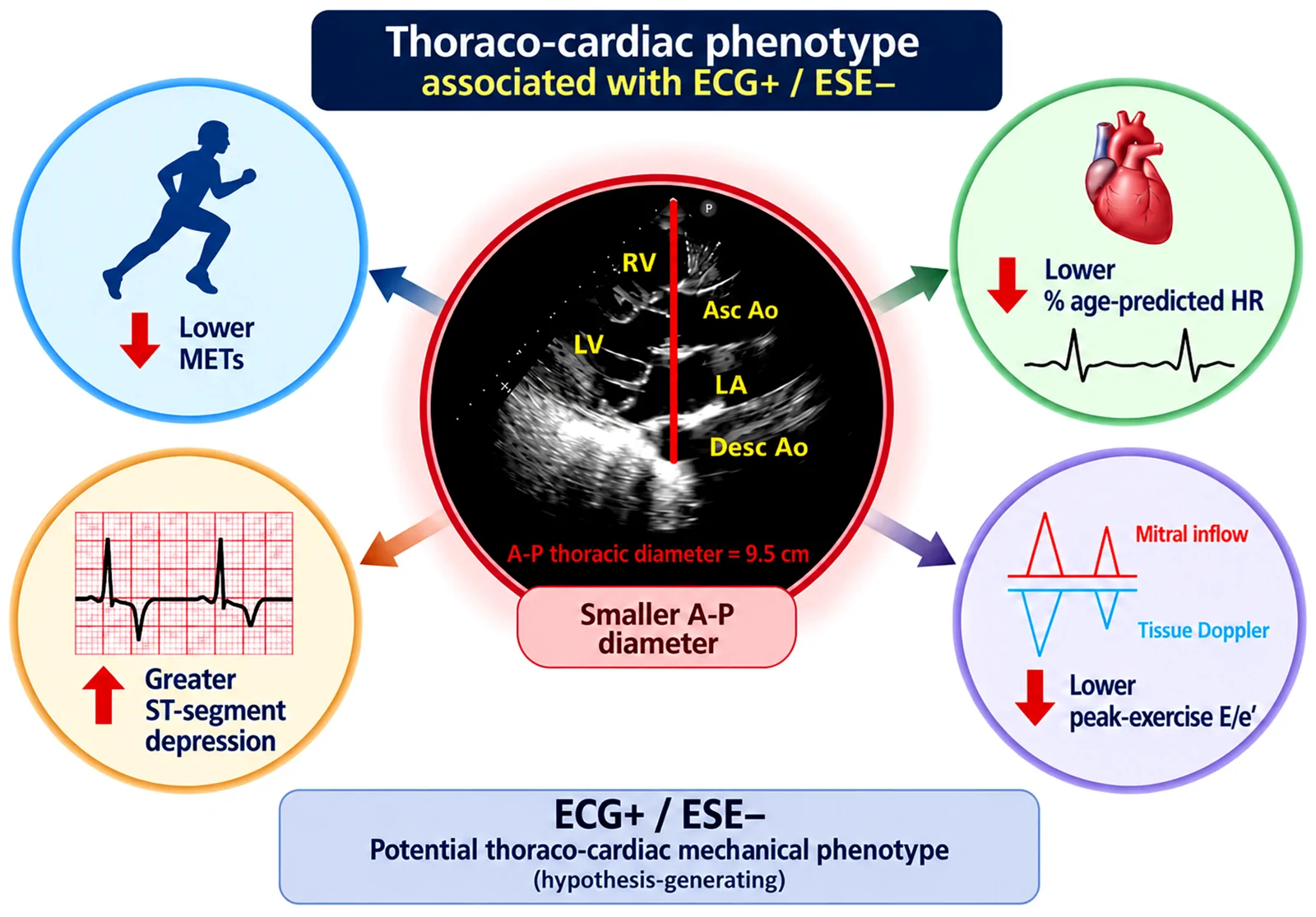
Schematic representation of the principal features associated with reduced A-P thoracic diameter in the present cohort. A-P, antero-posterior; ECG, electrocardiography; ESE, exercise stress echocardiography; HR, heart rate; LV, left ventricular; METs, metabolic equivalents. The schematic illustration was created with AI assistance (ChatGPT, GPT-5.6 Sol; OpenAI, San Francisco, CA, USA) and subsequently reviewed and edited by the authors to ensure scientific accuracy.

Accordingly, the combination of a narrow or concave thoracic configuration, MVP, reduced exercise capacity, attenuated chronotropic response, relatively low exercise filling-pressure estimates, and exercise-induced ST-segment depression despite preserved LV contractile reserve may identify a distinct phenotype in which thoraco-cardiac mechanical factors contribute to ECG–ESE discordance and functional limitation. The principal anatomical and functional features characterizing this proposed thoraco-cardiac phenotype are summarized in Figure 4.

From a practical perspective, recognition of this phenotype may help clinicians interpret exercise-induced ST-segment depression within the broader anatomical and functional context of the individual patient, particularly when ECG abnormalities coexist with preserved LV contractile reserve, low estimated filling pressures, and negative ESE. Thoracic geometry may therefore provide complementary phenotypic information in patients with ECG+/ESE− discordance.

Further validation should determine whether A-P thoracic diameter provides incremental discriminatory value beyond established clinical likelihood assessment and can contribute to more individualized use of downstream anatomical testing.

### 4.6. Study Limitations

Several limitations should be acknowledged. This was a prospective, single-center study conducted in a relatively selected population of symptomatic patients undergoing evaluation for suspected obstructive CAD. The cohort was predominantly female and included a relatively high proportion of individuals with MVP. However, enrollment was consecutive and was performed independently of MVP status or chest-wall configuration; therefore, the high prevalence of MVP reflects the characteristics of the enrolled cohort rather than preferential recruitment based on the phenotype of interest. Nevertheless, the particular demographic and phenotypic composition of the cohort may limit the generalizability of the present findings to populations with different demographic, anthropometric, cardiovascular risk, or mitral valve profiles.

Exercise was performed using a semi-supine bicycle protocol, and the relatively modest achieved workloads, peak heart rates, and rate-pressure products indicate that a true maximal central cardiovascular effort cannot be assumed in all participants. Peripheral muscular fatigue may have limited exercise performance, particularly in individuals not accustomed to cycling, potentially leading to exercise termination before central cardiovascular exhaustion. Moreover, treadmill exercise might have resulted in different workloads, hemodynamic responses, and electrocardiographic findings. Accordingly, the present exercise-related findings should be interpreted within the specific context of semi-supine bicycle ESE, and their reproducibility using other exercise modalities remains to be established.

A further limitation concerns the interpretation of exercise-induced ST-segment depression. Most patients classified as ECG-positive exhibited a pure upsloping ST-segment morphology (74.3%). Although upsloping ST-segment depression meeting the prespecified ≥ 1.5-mm criterion measured 80 ms after the J-point was considered positive in the present analysis, this morphology is less specific for myocardial ischemia and its diagnostic significance has historically been more controversial than that of horizontal or downsloping ST-segment depression [41]. Accordingly, the predominance of upsloping ST-segment changes should be considered when interpreting the prevalence and clinical significance of the ECG+/ESE− phenotype observed in our cohort.

Only 35 of 101 participants developed a positive exercise ECG, limiting the number of covariates that could be reliably incorporated into the multivariable model. A parsimonious modeling strategy was therefore adopted to reduce the risk of overfitting. Nevertheless, the wide confidence intervals surrounding some adjusted estimates, particularly those for MVP and exercise-induced hypertension, indicate limited statistical precision and potential model instability. Moreover, the large magnitude of some adjusted ORs, together with the strong inverse association observed for A-P thoracic diameter and its very high discriminatory performance, raises the possibility of sparse-data effects and quasi-complete separation, which may result in unstable maximum-likelihood estimates and inflation of effect sizes. Accordingly, the adjusted ORs should not be interpreted as precise estimates of the magnitude of association, and the multivariable findings should be regarded as exploratory pending confirmation in larger independent cohorts.

Although CCTA excluded obstructive epicardial CAD, coronary microvascular function and vasomotor abnormalities were not specifically assessed and therefore cannot be excluded as contributors to exercise-induced ST-segment depression. However, the observed exercise hemodynamic and functional profile did not provide indirect evidence strongly supporting this hypothesis. The present findings demonstrate an association between thoracic geometry and ECG+/ESE− discordance but cannot establish that the observed repolarization abnormalities were exclusively mechanically mediated.

The relationships between A-P thoracic diameter and exercise capacity, chronotropic response, magnitude of ST-segment depression, and peak-exercise E/e′ were cross-sectional and therefore cannot establish causality. Thoracic geometry may covary with other anthropometric, autonomic, positional, or cardiac mechanical characteristics that were not fully captured by the present analysis. Moreover, exercise E/e′ represents an indirect estimate of LV filling pressure and should not be considered equivalent to invasive hemodynamic assessment.

Echocardiographic measurements, particularly assessment of A-P thoracic diameter from the parasternal long-axis view, may also be subject to sampling variability. Individual anatomical variation and small differences in transducer orientation, imaging axis, or acquisition plane may influence the measured distance between the sternum and vertebral body. Although measurements were obtained using a standardized anatomical approach and the descending thoracic aorta as a landmark, residual measurement error cannot be excluded and should be considered when interpreting the observed associations and the data-derived A-P diameter threshold.

The ROC-derived A-P thoracic diameter threshold of ≤10.95 cm was identified and evaluated within the same study population. Although internal bootstrap validation demonstrated negligible optimism in the AUC, no independent external validation of the data-derived threshold was performed. Therefore, its sensitivity and specificity may not be directly generalizable to other populations. The proposed threshold should consequently be considered hypothesis-generating and should not currently be used to modify or withhold guideline-recommended coronary testing.

The study was not designed to evaluate long-term cardiovascular outcomes, downstream diagnostic resource utilization, or cost-effectiveness. Accordingly, whether incorporation of A-P thoracic diameter into contemporary clinical-likelihood assessment provides incremental discrimination or reclassification beyond established predictors—and whether such an approach can safely reduce unnecessary anatomical coronary testing—remains to be determined.

Larger prospective, multicenter studies incorporating external validation, assessment of coronary microvascular function, and longitudinal follow-up are required before thoracic geometry can be integrated into clinical decision algorithms.

## 5. Conclusions

In symptomatic patients undergoing evaluation for suspected CAD, exercise ECG positivity despite negative ESE and absence of obstructive CAD on CCTA was associated with a distinct thoraco-cardiac phenotype. Patients with ECG+/ESE− exhibited a narrower thoracic configuration and distinctive cardiac geometry, with more frequent MVP and a strong association between reduced A-P thoracic diameter and exercise ECG positivity. These findings support the hypothesis that restricted chest geometry and altered thoraco-cardiac and mitral apparatus mechanics may contribute to exercise-induced repolarization abnormalities despite preserved LV contractile reserve and without flow-limiting epicardial coronary disease.

A-P thoracic diameter represents a simple, radiation-free parameter that can be readily obtained during conventional TTE and may provide complementary phenotypic information when exercise ECG and stress echocardiographic findings are discordant. However, the proposed ≤10.95-cm threshold and the incremental value of thoracic geometry beyond established clinical-likelihood assessment require independent validation.

If confirmed in larger prospective multicenter cohorts, incorporation of A-P thoracic diameter into the integrated assessment of ECG+/ESE− discordance could refine interpretation of exercise-test findings, facilitate recognition of a potentially mechanically mediated phenotype, and support more individualized use of downstream anatomical coronary testing.

## Figures and Tables

**Table 1:** Baseline demographic, anthropometric, clinical, laboratory, and coronary and carotid imaging characteristics of the overall study population and according to exercise ECG response.

Variable	Overall Population (n = 101)	ECG+/ESE− (n = 35)	ECG−/ESE− (n = 66)	*p*-Value
Demographic and anthropometric characteristics				
Age, years	61 [55–67]	58 [51–67.5]	62.5 [56–67]	0.186
Female sex, n (%)	78 (77.2)	27 (77.1)	51 (77.3)	1.000
Male sex, n (%)	23 (22.8)	8 (22.9)	15 (22.7)	1.000
BSA, m^2^	1.66 [1.59–1.80]	1.67 [1.60–1.79]	1.65 [1.58–1.81]	0.509
BMI, kg/m^2^	21.9 ± 2.9	21.6 ± 3.3	22.0 ± 2.7	0.483
Latero-lateral thoracic diameter, cm	29 [28–31]	29 [28–30]	29 [28–31]	0.900
Antero-posterior thoracic diameter, cm	11.67 ± 1.43	10.15 ± 0.87	12.47 ± 1.07	**<0.001**
Modified Haller Index	2.50 [2.31–2.72]	2.86 [2.60–3.19]	2.34 [2.22–2.50]	**<0.001**
Cardiovascular risk factors				
Hypertension, n (%)	59 (58.4)	20 (57.1)	39 (59.1)	0.850
Diabetes mellitus, n (%)	3 (3.0)	2 (5.7)	1 (1.5)	0.275
Current/former smoking, n (%)	20 (19.8)	5 (14.3)	15 (22.7)	0.311
Dyslipidemia, n (%)	68 (67.3)	21 (60.0)	47 (71.2)	0.253
Cardiovascular history and comorbidities				
History of CAD, n (%)	2 (2.0)	1 (2.9)	1 (1.5)	1.000
Family history of premature CAD, n (%)	4 (4.0)	0 (0)	4 (6.1)	0.295
Estimated pre-test probability of obstructive CAD, %	9 [2–20]	7 [2–12]	12 [4–20]	0.20
Hypothyroidism, n (%)	7 (6.9)	1 (2.9)	6 (9.1)	0.417
COPD, n (%)	5 (5.0)	3 (8.6)	2 (3.0)	0.338
Previous functional ischemia assessment				
Previous exercise stress test equivocal for myocardial ischemia, n (%)	12 (11.9)	9 (25.7)	3 (4.5)	0.003
CCTA findings				
Normal/no CAD, n (%)	51 (50.5)	18 (51.4)	33 (50.0)	0.892
Mild non-obstructive coronary atherosclerosis/calcification, n (%)	50 (49.5)	17 (48.6)	33 (50.0)	0.892
Obstructive CAD ≥ 50%, n (%)	0 (0)	0 (0)	0 (0)	—
Carotid ultrasound findings				
Maximum carotid IMT, mm	1.10 ± 0.19	1.11 ± 0.19	1.10 ± 0.20	0.759
Carotid plaque, n (%)	25 (24.8)	8 (22.9)	17 (25.8)	0.813
Laboratory findings				
Hemoglobin, g/dL	13.6 ± 1.2	13.7 ± 1.0	13.6 ± 1.3	0.912
Glucose, mg/dL	91.1 ± 7.1	90.2 ± 6.1	91.6 ± 7.5	0.281
Creatinine, mg/dL	0.83 ± 0.15	0.82 ± 0.11	0.84 ± 0.16	0.603
LDL cholesterol, mg/dL	107.3 ± 32.1	105.0 ± 24.9	108.5 ± 35.4	0.755
Ongoing medical therapy				
Antiplatelet therapy, n (%)	11 (10.9)	1 (2.9)	10 (15.2)	0.092
Anticoagulant therapy, n (%)	1 (1.0)	0 (0)	1 (1.5)	1.000
Beta-blockers, n (%)	24 (23.8)	5 (14.3)	19 (28.8)	0.141
Antiarrhythmic drugs, n (%)	8 (7.9)	0 (0)	8 (12.1)	**0.048**
ACE inhibitors/ARBs, n (%)	29 (28.7)	10 (28.6)	19 (28.8)	1.000
Calcium channel blockers, n (%)	16 (15.8)	6 (17.1)	10 (15.2)	0.782
Diuretics, n (%)	7 (6.9)	2 (5.7)	5 (7.6)	1.000
Statin therapy, n (%)	35 (34.7)	9 (25.7)	26 (39.4)	0.193
Non-statin lipid-lowering therapy, n (%)	20 (19.8)	7 (20.0)	13 (19.7)	1.000

Data are presented as mean ± standard deviation, median [interquartile range], or number (percentage), as appropriate. Comparisons were performed between patients with a positive exercise ECG despite negative exercise stress echocardiography (ECG+/ESE−) and those with both negative exercise ECG and negative exercise stress echocardiography (ECG−/ESE−). Statistically significant *p*-values are shown in bold (*p* < 0.05). ACE, angiotensin-converting enzyme; ARB, angiotensin receptor blocker; BMI, body mass index; BSA, body surface area; CAD, coronary artery disease; CCTA, coronary computed tomography angiography; COPD, chronic obstructive pulmonary disease; ECG, electrocardiography; ESE, exercise stress echocardiography; IMT, intima-media thickness; LDL, low-density lipoprotein.

**Table 2:** Resting hemodynamic, electrocardiographic, and echocardiographic characteristics of the overall study population and according to exercise ECG response.

Variable	Overall Population (n = 101)	ECG+/ESE− (n = 35)	ECG−/ESE− (n = 66)	*p*-Value
Resting hemodynamic and ECG parameters				
Resting SBP, mmHg	112.4 ± 13.5	111.9 ± 14.0	112.7 ± 13.2	0.784
Resting DBP, mmHg	71.8 ± 7.4	71.1 ± 6.9	72.1 ± 7.7	0.516
Resting HR, bpm	72.2 ± 11.8	72.9 ± 13.4	71.8 ± 11.0	0.678
Resting oxygen saturation, %	98.1 ± 1.1	98.5 ± 1.1	98.0 ± 1.1	**0.044**
Resting intraventricular conduction				
Normal, n (%)	31 (30.7)	11 (31.4)	20 (30.3)	1.000
Incomplete RBBB, n (%)	48 (47.5)	16 (45.7)	32 (48.5)	0.836
LAFB, n (%)	2 (2.0)	1 (2.9)	1 (1.5)	1.000
Incomplete RBBB + LAFB, n (%)	16 (15.8)	5 (14.3)	11 (16.7)	1.000
Complete RBBB, n (%)	1 (1.0)	0 (0)	1 (1.5)	1.000
Complete RBBB + LAFB, n (%)	2 (2.0)	2 (5.7)	0 (0)	0.118
First-degree AV block + incomplete RBBB, n (%)	1 (1.0)	0 (0)	1 (1.5)	1.000
Resting echocardiographic parameters				
IVS thickness, mm	9.5 ± 2.0	9.0 ± 1.7	9.8 ± 2.2	0.063
PW thickness, mm	7.8 ± 1.1	7.5 ± 1.1	7.9 ± 1.1	**0.028**
LVEDD, mm	41.7 ± 4.1	41.6 ± 4.6	41.8 ± 3.8	0.808
RWT	0.38 ± 0.06	0.36 ± 0.06	0.38 ± 0.06	0.083
LVMi, g/m^2^	66.4 ± 17.2	60.9 ± 17.8	69.4 ± 16.2	**0.006**
LV geometry, systolic and diastolic function, and LA size				
Normal geometry, n (%)	72 (71.3)	29 (82.9)	43 (65.2)	0.069
Concentric remodeling, n (%)	23 (22.8)	5 (14.3)	18 (27.3)	0.212
Concentric hypertrophy, n (%)	2 (2.0)	0 (0)	2 (3.0)	0.543
Eccentric hypertrophy, n (%)	4 (4.0)	1 (2.9)	3 (4.5)	1.000
Resting E/A ratio	1.00 ± 0.29	1.09 ± 0.26	0.96 ± 0.29	**0.024**
Resting E/e′ ratio	6.03 ± 1.49	6.09 ± 1.65	6.00 ± 1.41	1.000
Resting LVEF, %	60.6 ± 3.2	60.3 ± 3.4	60.8 ± 3.1	0.491
LA antero-posterior diameter, mm	32.8 ± 5.4	29.1 ± 4.5	34.8 ± 4.7	**<0.001**
Mitral valve findings				
Mitral valve prolapse, n (%)	58 (57.4)	25 (71.4)	33 (50.0)	**0.038**
Trivial MR, n (%)	36 (35.6)	15 (42.9)	21 (31.8)	
Mild MR, n (%)	50 (49.5)	14 (40.0)	36 (54.5)	0.377 *
Mild-to-moderate MR, n (%)	15 (14.9)	6 (17.1)	9 (13.6)	
MAD, n (%)	32 (31.7)	11 (31.4)	21 (31.8)	0.968
MAD distance, mm **	6.7 ± 1.4	5.57 ± 0.75	7.23 ± 1.31	**<0.001**
Right-heart parameters				
RVIT, mm	27.5 ± 3.2	27.2 ± 3.9	27.6 ± 2.8	0.598
TAPSE, mm	24.4 ± 1.9	24.9 ± 2.0	24.1 ± 1.9	**0.008**
Resting sPAP, mmHg	25.6 ± 3.0	25.6 ± 2.9	25.5 ± 3.1	0.891

Data are presented as mean ± standard deviation or number (percentage), as appropriate. Comparisons were performed between patients with a positive exercise ECG despite negative exercise stress echocardiography (ECG+/ESE−) and those with both negative exercise ECG and negative exercise stress echocardiography (ECG−/ESE−). Statistically significant *p*-values are shown in bold (*p* < 0.05). * *p*-value refers to the overall between-group comparison of the distribution of resting MR severity across the three categories (trivial, mild, and mild-to-moderate). ** MAD distance was assessed only in patients with mitral annular disjunction. AV, atrioventricular; DBP, diastolic blood pressure; ECG, electrocardiography; ESE, exercise stress echocardiography; HR, heart rate; IVS, interventricular septum; LA, left atrial; LAFB, left anterior fascicular block; LVEDD, left ventricular end-diastolic diameter; LVEF, left ventricular ejection fraction; LVMi, left ventricular mass index; MAD, mitral annular disjunction; MR, mitral regurgitation; MVP, mitral valve prolapse; PW, posterior wall; RBBB, right bundle branch block; RVIT, right ventricular inflow tract; RWT, relative wall thickness; SBP, systolic blood pressure; sPAP, systolic pulmonary artery pressure; TAPSE, tricuspid annular plane systolic excursion.

**Table 3:** Exercise capacity, hemodynamic, electrocardiographic, arrhythmic, symptomatic, and echocardiographic responses during exercise stress echocardiography in the overall study population and according to exercise ECG response.

Variable	Overall Population (n = 101)	ECG+/ESE− (n = 35)	ECG−/ESE− (n = 66)	*p*-Value
Exercise capacity and hemodynamic response				
Maximum workload, W	82.4 ± 26.6	75.0 ± 20.1	86.4 ± 28.8	0.039
Exercise capacity, METs	6.13 ± 1.19	5.74 ± 1.25	6.32 ± 1.11	0.014
Maximum HR, bpm	124.8 ± 15.8	130.7 ± 13.4	121.6 ± 16.2	0.015
Maximum predicted HR achieved, %	77.1 ± 8.2	79.6 ± 5.7	75.7 ± 9.0	0.077
Peak exercise oxygen saturation, %	98.0 ± 1.3	98.2 ± 1.2	97.8 ± 1.3	0.231
Peak exercise SBP, mmHg	160.2 ± 19.9	163.0 ± 22.2	158.8 ± 18.7	0.317
Peak exercise DBP, mmHg	84.1 ± 10.4	84.0 ± 9.3	84.1 ± 11.0	0.935
Rate-pressure product, mmHg × bpm	19,914 ± 3664	20,684 ± 3231	19,506 ± 3835	0.107
Exercise-induced hypertension, n (%)	20 (19.8)	14 (40.0)	6 (9.1)	**<0.001**
Exercise-induced ST-segment findings				
Maximum inferolateral ST-segment depression, mV	0.08 ± 0.04	0.127 ± 0.032	0.061 ± 0.020	<0.001
Positive exercise ECG according to prespecified criteria, n (%)	35 (34.7)	35 (100)	0 (0)	—
Predominant exercise ST-segment morphology				
Pure upsloping, n (%)	82 (81.2)	26 (74.3)	56 (84.8)	0.284
Non-pure upsloping/mixed, n (%)	12 (11.9)	5 (14.3)	7 (10.6)	0.748
Downsloping, n (%)	6 (5.9)	4 (11.4)	2 (3.0)	0.178
Horizontal, n (%)	1 (1.0)	0 (0)	1 (1.5)	1.000
Exercise test-related arrhythmias				
No arrhythmias, n (%)	52 (51.5)	17 (48.6)	35 (53.0)	0.682
Low arrhythmic burden (index 1), n (%)	35 (34.7)	12 (34.3)	23 (34.8)	1.000
Moderate arrhythmic burden (index 2), n (%)	9 (8.9)	3 (8.6)	6 (9.1)	1.000
Complex arrhythmic burden (index 3), n (%)	5 (5.0)	3 (8.6)	2 (3.0)	0.338
Exercise-related symptoms				
Any symptoms, n (%)	19 (18.8)	4 (11.4)	15 (22.7)	0.193
Dyspnea, n (%)	17 (16.8)	3 (8.6)	14 (21.2)	0.162
Palpitations, n (%)	2 (2.0)	1 (2.9)	1 (1.5)	1.000
Peak exercise echocardiographic parameters				
Peak exercise E/A ratio	1.11 ± 0.32	1.18 ± 0.29	1.07 ± 0.32	0.050
Peak exercise E/e′ ratio	7.42 ± 2.61	7.58 ± 3.28	7.33 ± 2.21	0.761
Peak exercise LVEF, %	71.8 ± 3.7	72.0 ± 3.7	71.7 ± 3.7	0.555
Peak exercise sPAP, mmHg	42.4 ± 7.2	42.9 ± 7.1	42.1 ± 7.2	1.000
Peak exercise mitral regurgitation				
Trivial, n (%)	35 (34.7)	15 (42.9)	20 (30.3)	0.272
Mild, n (%)	42 (41.6)	12 (34.3)	30 (45.5)	0.298
Mild-to-moderate, n (%)	17 (16.8)	5 (14.3)	12 (18.2)	0.782
Moderate, n (%)	7 (6.9)	3 (8.6)	4 (6.1)	0.691
Changes from rest to peak exercise				
Δ oxygen saturation, %	−0.18 ± 1.19	−0.29 ± 0.96	−0.12 ± 1.30	0.587
Δ HR, bpm	52.6 ± 15.1	57.9 ± 16.2	49.8 ± 13.8	0.012
Δ SBP, mmHg	47.9 ± 18.6	51.1 ± 18.7	46.1 ± 18.4	0.199
Δ DBP, mmHg	12.3 ± 9.5	12.9 ± 8.7	12.0 ± 10.0	0.629
Δ E/A ratio	0.10 ± 0.26	0.09 ± 0.22	0.11 ± 0.28	0.701
Δ E/e′ ratio	1.39 ± 1.86	1.50 ± 2.30	1.33 ± 1.59	0.997
Δ LVEF, %	11.2 ± 2.2	11.7 ± 2.4	10.9 ± 2.1	0.081
Δ sPAP, mmHg	16.8 ± 6.0	17.2 ± 6.3	16.6 ± 5.9	0.685

Data are presented as mean ± standard deviation or number (percentage), as appropriate. Comparisons were performed between patients with a positive exercise ECG despite negative exercise stress echocardiography (ECG+/ESE−) and those with both negative exercise ECG and negative exercise stress echocardiography (ECG−/ESE−). ST-segment morphology was classified in all participants according to the predominant configuration observed during exercise, irrespective of whether the magnitude of ST-segment depression fulfilled the prespecified criteria for exercise ECG positivity; therefore, the reported morphology categories should not be interpreted as indicating a positive exercise ECG. Statistically significant *p*-values are shown in bold (*p* < 0.05). Exercise-induced hypertension was defined as a peak exercise systolic blood pressure ≥ 210 mmHg in men or ≥190 mmHg in women. Exercise-related arrhythmic burden was categorized using a four-level index: 0, no arrhythmias; 1, low burden, characterized predominantly by rare or occasional isolated supraventricular or ventricular premature beats; 2, moderate burden, characterized by more frequent ectopy and/or couplets; and 3, complex burden, including complex supraventricular or ventricular ectopy such as bigeminy, triplets, quadruplets, or runs of supraventricular tachycardia. Δ indicates the change from rest to peak exercise, calculated as peak-exercise value minus resting value. DBP, diastolic blood pressure; ECG, electrocardiography; ESE, exercise stress echocardiography; HR, heart rate; LVEF, left ventricular ejection fraction; METs, metabolic equivalents; MR, mitral regurgitation; SBP, systolic blood pressure; SPB, supraventricular premature beat; sPAP, systolic pulmonary artery pressure; SVT, supraventricular tachycardia; VPB, ventricular premature beat.

**Table 4:** Univariable and multivariable logistic regression analysis of factors associated with a positive exercise ECG despite negative exercise stress echocardiography.

Variable	Univariable OR	95% CI	*p*-Value	Adjusted OR	95% CI	*p*-Value
PTP of obstructive CAD, per 1% increase	0.92	0.81–1.04	0.195	—	—	—
Exercise-induced hypertension	6.67	2.27–19.59	**<0.001**	5.74	0.64–51.52	0.119
Mitral valve prolapse	2.50	1.04–6.01	**0.041**	8.50	1.07–67.75	**0.043**
A-P thoracic diameter, per 1-cm increase	0.036	0.009–0.149	**<0.001**	0.026	0.005–0.143	**<0.001**

Odds ratios (ORs) and corresponding 95% confidence intervals (CIs) are reported for factors evaluated in relation to exercise ECG positivity despite negative exercise stress echocardiography (ECG+/ESE−). Adjusted ORs derive from the parsimonious multivariable logistic regression model. The multivariable model included exercise-induced hypertension, MVP, and A-P thoracic diameter; PTP was evaluated only in univariable analysis and was therefore not assigned an adjusted estimate. A-P thoracic diameter was modeled as a continuous variable, with the OR expressed per 1-cm increase. PTP was expressed per 1% increase. Statistically significant *p*-values are shown in bold (*p* < 0.05). A-P, antero-posterior; CAD, coronary artery disease; CI, confidence interval; ECG, electrocardiography; ESE, exercise stress echocardiography; MVP, mitral valve prolapse; OR, odds ratio; PTP, pre-test probability.

## Data Availability

The raw data supporting the findings of this study will be made openly accessible through Zenodo (https://zenodo.org; accssed on 5 October 2026).

## Supplementary Materials

The following supporting information can be downloaded at: https://www.mdpi.com/article/10.3390/jcm15218292/s1, File S1: Full study protocol of the prospective MVP&ESE project; File S2: Final Ethics Committee documentation for the MVP&ESE project issued by the Comitato Etico Territoriale Lombardia 5 on 3 November 2025.
