# Supplementary material for: When Exercise ECG and Stress Echocardiography Disagree: Thoracic Geometry as a Potential Determinant of Exercise-Induced ST-Segment Abnormalities
Source: J Clin Med. 2026 Oct 24;15(21):8292. doi: 10.3390/jcm15218292 (PMC13763842; doi:10.3390/jcm15218292)
Supplement: Supplementary file 1 [file jcm-15-08292-s001.zip › Supplementary Material File S1.pdf]

THE INFLUENCE OF CHEST WALL CONFORMATION ON EXERCISE TOLERANCE IN A PROSPECTIVE COHORT OF SYMPTOMATIC INDIVIDUALS WITH MITRAL VALVE PROLAPSE UNDERGOING EXERCISE STRESS ECHOCARDIOGRAPHY

ACRONYME: Project "MVP&ESE"

Principal Investigator: *Andrea Sonaglioni M.D. (P.I.)*

Institutions: *MultiMedica*

Corresponding Author: *Andrea Sonaglioni M.D.*
Division of Cardiology
Multimedica - San Giuseppe H
andrea.sonaglioni@multimedica.it

CONFIDENTIAL

This document is confidential and the property of individual. No part of it may be transmitted, reproduced, published, or used by other persons without prior written authorization from the author.

List of Abbreviations

2D, two-dimensional; A-P, antero-posterior; BMI, body mass index; BSA, body surface area; CAD, coronary artery disease; CI, confidence intervals; DBP, diastolic blood pressure; ECG, electrocardiogram; ESE, exercise stress echocardiography *GLS, global longitudinal strain; HR, heart rate; IVC, inferior vena cava; LA, left atrial; L-L, latero-lateral; LAVi, left atrial volume indexed; LV, left ventricular; LVEDVi, left ventricular end-diastolic volume indexed; LVEF, left ventricular ejection fraction; LVESVi, left ventricular end-systolic volume indexed; LVFP, left ventricular filling pressure; LVMi, left ventricular mass index; MACE, major adverse cardiovascular events; MHI, modified Haller index; MR, mitral regurgitation; MVP, mitral valve prolapse; PE, pectus excavatum; RV, right ventricular; RVIT, right ventricular inflow tract; RWT, relative wall thickness; SBP, systolic blood pressure; sPAP, systolic pulmonary artery pressure; STE, speckle tracking echocardiography; TAPSE, tricuspid annular plane systolic excursion; TRV, tricuspid regurgitation velocity; TTE, transthoracic echocardiography.*

Table of Contents

1 INTRODUCTION... 4

2 STUDY OBJECTIVES... 5

2.1 PRIMARY OBJECTIVE 5
2.2 SECONDARY OBJECTIVE 5

3 PATIENTS AND METHODS 5

3.1 STUDY DESIGN 5
3.1.1 General Design 5
3.1.2 Primary Outcome Variable... *5*
3.1.3 Secondary Outcome Variables... *5*
3.2 SUBJECT SELECTION AND WITHDRAWAL... 5
3.2.1 Inclusion Criteria... 6
3.2.2 Exclusion Criteria... 6
3.3 STUDY PROCEDURES... 6
3.3.1 *Clinical data* 6
3.3.2 *Modified Haller index assessment*... 7
3.3.3 *Resting transthoracic echocardiography*... 7
3.3.4 *Speckle tracking echocardiography*... .7
3.3.5 *Exercise stress echocardiography* 8
3.5 Statistical Plan... 9
3.5.1 Sample Size Determination... *9*
3.5.2 Statistical Methods... *9,*10

4 DATA HANDLING AND RECORD KEEPING... 10

5 **PUBLICATION PLAN 10**

6 REFERENCES... 11-13

1 Introduction

This document is a clinical research protocol and the described study will be conducted in accordance with the ethical standards of our Institutional Research Committee and with the 1964 Helsinki Declaration and its later amendments or comparable ethical standards.

Mitral valve prolapse (MVP) is a common valvular disorder that affects 2-3% of the general population [1]. It is defined as the systolic displacement of one or both leaflets of at least 2 mm above the annular plane into the left atrium, visualized from the parasternal long-axis view on resting transthoracic echocardiography (TTE) [2].

Even if MVP is generally considered a benign condition, a subset of individuals may be affected by complex ventricular arrhythmias, mitral regurgitation (MR), infective endocarditis, stroke, atrial fibrillation and even sudden cardiac death [3-5].

Due to the multidimensional nature of this disease, risk stratification should carefully incorporate markers of left ventricular dysfunction, arrhythmic burden, and exercise capacity.

Several MVP individuals have exercise intolerance [6]. The underlying mechanisms of exercise intolerance in such individuals are still not completely understood. Autonomic dysfunction [7], hyperadrenergic state [8], reduced left ventricular filling [9], limited right ventricular contractile reserve [10], catecholamine excess [11] or combinations of these conditions have been reported as potential mechanisms of exercise intolerance in MVP individuals.

MVP symptoms are frequently disproportionate to objective findings derived from exhaustive clinical, laboratory and echocardiographic investigation. Indeed, the great majority of MVP individuals are diagnosed with mild or moderate MR, that should have a modest impact on exercise tolerance.

Despite the prevalence of MVP in the general population and the known exercise intolerance, there is little literature about the exercise capacity of MVP patients. To date, only a few studies have assessed functional capacity during exercise in MVP individuals vs. symptomatic individuals without MVP by exercise stress echocardiography (ESE) [10,12] or cardiopulmonary exercise test (CPET) [13-15]. These authors ascribed the effort intolerance, detected in MVP individuals, to several factors, such as chronotropic incompetence, abnormal pulmonary vascular reserve, and limited right ventricular contractile reserve, and not simply because of exercise-induced MR or pulmonary hypertension. However, they did not consider the possible influence of anthropometrics, particularly the chest wall conformation, on exercise tolerance of MVP individuals.

Literature data suggest a strong relationship between MVP and various forms of chest wall abnormalities, such as pectus excavatum (PE), pectus carinatum, straight back syndrome and scoliosis [16]. During the last few years, our study group has conceived the modified Haller index (MHI), a nonradiological method to quantify the chest wall conformation without ionizing radiation exposure. This index is obtained by dividing the latero-lateral (L-L) thoracic diameter (measured by using a rigid ruler coupled to a level) to the antero-posterior (A-P) thoracic diameter (measured from the echocardiographic parasternal long-axis view, as the distance between the true apex of the imaging sector and the posterior wall of the descending aorta, visualized behind the left atrium) [17]. A MHI >2.5 is diagnostic of pectus excavatum or concave-shaped chest wall conformation, whereas a MHI ≤ 2.5 identifies individuals with a normal chest shape conformation [18].

We have previously demonstrated that MVP individuals are commonly found a concave-shaped chest wall conformation and that MVP individuals with MHI >2.5, despite reduced exercise tolerance and impaired myocardial deformation indices at basal level, have a low prevalence of major adverse cardiovascular events (MACE) over a medium-term follow-up [19,20].

We hypothesized that MVP individuals, who are commonly found with various degrees of anterior chest wall deformity, might have a significantly lower exercise tolerance compared to healthy controls without MVP. Moreover, we hypothesized that peak-exercise MR degree might be significantly less severe in MVP individuals with MHI >2.5 than in those with MHI ≤2.5. Accordingly, the present study has been primarily designed to assess the influence of chest wall conformation, noninvasively assessed by MHI, on exercise tolerance assessed by ESE, in a consecutive cohort of symptomatic MVP individuals without severe MR, compared to a control group of symptomatic individuals without MVP, matched by age, and sex. Secondly, we aimed at investigating the effect of MHI on peak-exercise MR degree, assessed by ESE, in the same cohort of MVP individuals.

2 Study Objectives

2.1 Primary Objective

To evaluate if exercise tolerance, assessed by ESE, is significantly impaired in symptomatic MVP individuals in comparison to symptomatic controls without MVP, matched by age and sex.

2.2 Secondary Objective

To evaluate the relationship between the chest wall conformation, noninvasively assessed by MHI, and peak-exercise MR degree, quantified by ESE, in the same cohort of MVP individuals.

3 Patients and Methods

3.1 Study Design

3.1.1 General Design

Observational, case-control, prospective, monocentric study. Study duration: 6 months.

3.1.2 Primary Outcome Variable

The present study primarily aims at evaluating if exercise tolerance (assessed by the peak exercise watts on ESE) is significantly reduced in symptomatic MVP individuals, in comparison to symptomatic controls without MVP, matched by age and sex.

3.1.3 Secondary outcome Variable

The present study also aims at assessing the relationship between MHI and peak-exercise MR degree, quantified by the peak-exercise effective regurgitant orifice area (EROA) [21] on ESE, in the same cohort of MVP individuals.

3.2 Subject Selection and Withdrawal

3.2.1 Inclusion Criteria concerning MVP individuals

MVP, defined as a systolic billowing of one or both mitral leaflets >2 mm above the mitral annulus in the parasternal long-axis view [2]; mild or moderate MR according to the AHA/ACC recommendations for the assessment of valvular regurgitation [22], preserved left ventricular ejection fraction (LVEF) (≥55%) [23] and absence of severe pulmonary hypertension [tricuspid regurgitation velocity (TRV) >3.4 m/s)] [24] on resting TTE; symptoms, such as resting and/or exercise-induced dyspnea, chest pain, and/or palpitations; individuals with reduced exercise tolerance.

3.2.2 Exclusion Criteria concerning MVP individuals

Age <18 yrs; absence of symptoms such as resting and/or exercise-induced dyspnea, chest pain, and/or palpitations; history of coronary artery disease (CAD); resting severe primary MR, concomitant more than mild aortic stenosis or regurgitation, LVEF <50% and severe pulmonary hypertension (TRV >3.4 m/s) on resting TTE; atrial fibrillation on resting electrocardiogram (ECG); asymptomatic status; hemodynamic instability; relevant comorbidities; poor echocardiographic acoustic windows; inability to perform physical exercise~~; lack of consent~~.

3.2.3 Inclusion Criteria concerning controls

Individuals without MVP and without relevant cardiovascular disorders, respiratory diseases and/or renal diseases, matched by age and sex, undergoing ESE for symptoms, such as resting and/or exercise-induced dyspnea, chest pain, and/or palpitations.

3.2.4 Exclusion Criteria concerning controls

Age <18 yrs; absence of symptoms such as resting and/or exercise-induced dyspnea, chest pain, and/or palpitations; relevant cardiovascular, pulmonary or renal comorbidities; hemodynamic instability; poor or inadequate echocardiographic acoustic windows; inability to perform physical exercise.~~; individual's refusal to participate in the present clinical research.~~

Both groups of individuals (MVP individuals and controls) will be selected from the Cardiology Outpatient Clinic of the Ospedale San Giuseppe MultiMedica., where they are referred by the family doctor to perform conventional TTE because of symptoms, such as resting and/or exercise-induced dyspnea, chest pain, and/or palpitations.

3.3 Study Procedures

3.3.1 Clinical data

Following parameters will be recorded at basal evaluation: age, gender, body surface area (BSA), body mass index (BMI), systolic blood pressure (SBP) and diastolic blood pressure (DBP), presence of cardiovascular risk factors (smoking, hypertension, diabetes,

dyslipidemia), pharmacological treatment (if any). ECG data will include cardiac rhythm, heart rate, left ventricular (LV) depolarization and repolarization pattern.

Each patient will undergo resting blood pressure measurement with a semiautomatic sphygmomanometer (Omron 705IT; Omron, Kyoto, Japan), electrocardiogram (ECG), MHI assessment, resting TTE implemented with speckle tracking echocardiography (STE) and subsequently ESE. ESE will be performed during the first visit in the same Cardiology Outpatient Clinic of the Ospedale San Giuseppe MultiMedica.

3.3.2 Modified Haller index assessment

MHI measurement has been developed in our echo lab and has been previously validated in a comparative study of transthoracic ultrasound and chest X-rays [17]. It is calculated by dividing the maximum L-L external thoracic diameter by the minimum A-P internal thoracic diameter. Both thoracic diameters are measured at the end of inspiration. This approach yields values of chest shape comparable to those obtainable through chest X-rays, without exposing the subject to radiations [17]. Considering the PE definition based on a Haller index value >2.5 [18], a normal MHI should be ≤2.5, whereas a concave-shaped chest wall is defined by an MHI>2.5.

3.3.3 Resting transthoracic echocardiography

All resting echocardiograms will be performed with the participant in left-lateral decubitus, and parameters measured according to the Recommendations of the American Society of Echocardiography and the European Association of Cardiovascular Imaging [25,26]. Echocardiographic measurements will be indexed to BSA.

Following conventional two-dimensional (2D) echoDoppler parameters will be recorded: the aortic root and ascending aorta diameters by using the “leading edge-to-leading edge” convention; the relative wall thickness (RWT), calculated with the formula RWT = 2 posterior wall thickness/LV internal diameter at end-diastole; the left ventricular mass index (LVMi), calculated by the Devereux’s formula; the left ventricular end-diastolic volume index (LVEDVi) and the left ventricular end-systolic volume indexed (LVESVi); the left ventricular ejection fraction (LVEF), estimated with the biplane modified Simpson’s method, as index of LV systolic function [25]; the left atrial volume index (LAVi); the right ventricular inflow tract (RVIT); the tricuspid annular plane systolic excursion (TAPSE), as index of right ventricular (RV) systolic function; finally, the inferior vena cava (IVC) diameter during normal respiration.

Doppler measurements will include the E/A ratio and the E/average e’ ratio, as indices of LV diastolic function and left ventricular filling pressure (LVFP), respectively [26]. Systolic pulmonary artery pressure (sPAP) will be derived by the modified Bernoulli equation, where sPAP = 4 × $(TRV)^2$ + right atrial pressure [23]. The latter will be estimated from IVC diameter and collapsibility.

MVP will be defined as a systolic billowing of one or both mitral leaflets >2 mm above the mitral annulus in the long-axis parasternal view [2]. A maximal leaflet thickness of ≥5 mm will be defined as classic MVP and otherwise was defined as nonclassic MVP. MR degree will be assessed according to the AHA/ACC recommendations for the assessment of valvular regurgitation [22].

3.3.4 Speckle-tracking echocardiography

Immediately after conventional TTE, high-resolution speckle-tracking strain analyses will be performed on 2D images in the following order: apical four-chamber, two-chamber and three-chamber views for LV longitudinal strain, using the automated function imaging and the Q-Analysis module [27]. According to Philips QLAB software, LV wall will be divided into seven segments in each apical view. LV peak systolic strain is defined as the point of maximal systolic shortening for each segment. A single bull's-eye summary for LV-global longitudinal strain (GLS) will be obtained, presenting the analysis for each segment along with the assessment of global LV strain. Early peak diastolic strain rate will be derived from longitudinal measurements [27]. Absolute values superior to 20% for LV-GLS [23] will be considered as the cut-off values of normality for comparison.

3.3.5 Exercise stress echocardiography

Patients will undergo incremental exercise test on a semisupine bicycle ergometer (Ergoselect 1200 ELP; Ergoline, Bitz, Germany), with initial workload set at 25 W, increased by 25 W every 2 min [28].

A 12-lead ECG will be continuously monitored. Heart rate (HR), blood pressure, and peripheral arterial oxygen saturation (SaO2) will be measured at rest, every 2 min during the test, at peak exercise, and during recovery.

All stress echocardiograms will be performed by the same cardiologist using a commercially available Philips Sparq ultrasound machine (Philips, Andover, Massacchusetts, USA) with a 2.5 MHz transducer, with the patient positioned on a semirecumbent cycle ergometer.

All conventional echo Doppler measurements will be performed according to the recommendations of the American Society of Echocardiography and the European Association of CV Imaging [25,26].

The following echo Doppler parameters will be collected at rest, during the second minute of each stage, and at peak exercise: LV ejection fraction (LVEF), LV diastolic function assessed by the E/A ratio and the average E/e' ratio, degree of MR severity, tricuspid annular plane systolic excursion, and finally systolic pulmonary artery pressure (sPAP).

For each measurement, at least three cardiac cycles will be averaged.

A hypercontractile response in all wall segments will be considered normal (negative ESE), regardless of exercise ECG results. Ischemia will be defined as new or worsened wall motion abnormalities during stress as indicated by an increase in wall motion score ≥1 grade in ≥2 segments (positive ESE) [28].

Degree of MR severity will be assessed by evaluating the regurgitant jet area using color flow Doppler (qualitative assessment) and by quantitative methods, such as vena contracta and EROA [21]. Exercise-induced severe primary MR will be diagnosed in case of an increase in EROA ≥0.13 cm^2 during exercise, according to previous studies [29].

***Table 1** summarizes all the visits and procedures, detailed with timing, planned for each individual included in the present study.*

Table 1: Schedule of Study Procedures

**Study Phase**	**Screening**
**Visit number**	**Basal evaluation**
~~Informed Consent~~	~~X~~
~~Review Inclusion/Exclusion Criteria~~	~~X~~
Demographics/Medical History^	X
Anthropometrics: BSA, BMI, MHI^	X
Physical Examination^	X
Vital Signs: BP, HR^	X
ECG^	X
Resting TTE^	X
STE*	X
ESE^	X
Review Inclusion/Exclusion Criteria	X
Informed Consent	X

^*Normal clinical practice; *Procedure in addition to normal clinical practice*

3.5 Statistical Plan

3.5.1 Sample Size Determination

A sample size of 31 symptomatic MVP individuals and 31 symptomatic controls without MVP, matched by age and sex, reach 80% of statistical power to detect a 25-point difference in the watts reached at peak exercise by the two study groups (i.e. 100 vs 125 watts, respectively) [20], assuming a pooled standard deviation of 35 units, using a two-sided equal-variance t-test with a level of significance (alpha) of 5%.

3.5.2 Statistical Methods

Normality of the distribution of continuous variables will be tested using the Kolmogorov–Smirnov test. Normally distributed variables will be summarized as mean ± standard deviation, while not normally distributed data will be expressed as median with first and third quartiles. The difference between means will be estimated by an independent two-tailed t-test for normally distributed variables, whereas the Mann-Whitney test will be used to compare the medians from paired samples for non-normally distributed variables. Categorical variables will be compared using chi-squared test.

Univariate and multivariate logistic regression analyses will be performed for identifying the independent predictors of exercise intolerance, defined by <100 watts reached at peak exercise [20]. For each variable investigated, correspondent odds ratios with 95% confidence intervals (CIs) will be calculated. Only the variables with $p < 0.10$

on univariate analysis will be thereafter included in the multivariate logistic regression model.

Relationships between MHI and peak exercise EROA and/or between MHI and resting LV-GLS in MVP individuals will be assessed by using Pearson or Spearman correlations. Statistical analysis will be performed with SPSS version 28 (SPSS Inc., Chicago, Illinois, USA), with two-tailed P values below 0.05 deemed statistically significant.

4 Data Handling and Record Keeping

Data to be collected**:** demographic, anthropometric, clinical, biochemical and echocardiographic data (as already listed in the Study Procedures section).

All electronic data will be collected from the Hospital repository and will be reported, as pseudonymized records, in a Excel DB, at baseline and during each follow-up visits.

5 Publication Plan

Only aggregate data without individually identifiable information will be published.

We intend to publish the present study within the next 12 months.

6 References

1. Freed LA, Levy D, Levine RA, Larson MG, Evans JC, Fuller DL, Lehman B, Benjamin EJ. Prevalence and clinical outcome of mitral-valve prolapse. N Engl J Med. 1999 Jul 1;341(1):1-7. doi: 10.1056/NEJM199907013410101. PMID: 10387935.
2. Parwani P, Avierinos JF, Levine RA, Delling FN. Mitral Valve Prolapse: Multimodality Imaging and Genetic Insights. Prog Cardiovasc Dis. 2017 Nov-Dec;60(3):361-369. doi: 10.1016/j.pcad.2017.10.007. Epub 2017 Nov 6. PMID: 29122631; PMCID: PMC5805573.
3. Devereux RB, Hawkins I, Kramer-Fox R, Lutas EM, Hammond IW, Spitzer MC, Hochreiter C, Roberts RB, Belkin RN, Kligfield P, et al. Complications of mitral valve prolapse. Disproportionate occurrence in men and older patients. Am J Med. 1986 Nov;81(5):751-8. doi: 10.1016/0002-9343(86)90339-6. PMID: 3776983.
4. Düren DR, Becker AE, Dunning AJ. Long-term follow-up of idiopathic mitral valve prolapse in 300 patients: a prospective study. J Am Coll Cardiol. 1988 Jan;11(1):42-7. doi: 10.1016/0735-1097(88)90164-7. PMID: 3335704.
5. Basso C, Iliceto S, Thiene G, Perazzolo Marra M. Mitral Valve Prolapse, Ventricular Arrhythmias, and Sudden Death. Circulation. 2019 Sep 10;140(11):952-964. doi: 10.1161/CIRCULATIONAHA.118.034075. Epub 2019 Sep 9. PMID: 31498700.
6. Gati S, Malhotra A, Sharma S. Exercise recommendations in patients with valvular heart disease. Heart. 2019 Jan;105(2):106-110. doi: 10.1136/heartjnl-2018-313372. Epub 2018 Sep 27. PMID: 30262455.
7. Bashore TM, Grines CL, Utlak D, Boudoulas H, Wooley CF. Postural exercise abnormalities in symptomatic patients with mitral valve prolapse. J Am Coll Cardiol. 1988 Mar;11(3):499-507. doi: 10.1016/0735-1097(88)91523-9. PMID: 2830325.

8. Styres KS. The phenomenon of dysautonomia and mitral valve prolapse. J Am Acad Nurse Pract. 1994 Jan;6(1):11-5. doi: 10.1111/j.1745-7599.1994.tb00888.x. PMID: 8003350.
9. Iskandrian AS. Exercise left ventricular performance in patients with mitral valve prolapse. Herz. 1988 Aug;13(4):243-8. PMID: 3169678.
10. Utsunomiya H, Hidaka T, Susawa H, Izumi K, Harada Y, Kinoshita M, Itakura K, Masada K, Kihara Y. Exercise-Stress Echocardiography and Effort Intolerance in Asymptomatic/Minimally Symptomatic Patients With Degenerative Mitral Regurgitation Combined Invasive-Noninvasive Hemodynamic Monitoring. Circ Cardiovasc Imaging. 2018 Sep;11(9):e007282. doi: 10.1161/CIRCIMAGING.117.007282. PMID: 30354676.
11. Pasternac A, Tubau JF, Puddu PE, Król RB, de Champlain J. Increased plasma catecholamine levels in patients with symptomatic mitral valve prolapse. Am J Med. 1982 Dec;73(6):783-90. doi: 10.1016/0002-9343(82)90758-6. PMID: 6216809.
12. Coisne A, Levy F, Malaquin D, Richardson M, Quéré JP, Montaigne D, Tribouilloy C. Feasibility of Doppler hemodynamic evaluation of primary and secondary mitral regurgitation during exercise echocardiography. Int J Cardiovasc Imaging. 2015 Feb;31(2):291-9. doi: 10.1007/s10554-014-0550-5. Epub 2014 Oct 18. PMID: 25326411.
13. Koutlianos NA, Kouidi EJ, Metaxas TI, Deligiannis AP. Non-invasive cardiac electrophysiological indices in soccer players with mitral valve prolapse. Eur J Cardiovasc Prev Rehabil. 2004 Oct;11(5):435-41. PMID: 15616419.
14. Huang MH, Tuan SH, Tsai YJ, Huang WC, Huang TC, Chang ST, Lin KL. Comparison of the Results of Cardiopulmonary Exercise Testing between Healthy Peers and Pediatric Patients with Different Echocardiographic Severity of Mitral Valve Prolapse. Life (Basel). 2023 Jan 21;13(2):302. doi: 10.3390/life13020302. PMID: 36836660; PMCID: PMC9958875.
15. Chung JH, Tsai YJ, Lin KL, Weng KP, Huang MH, Chen GB, Tuan SH. Comparison of Cardiorespiratory Fitness between Patients with Mitral Valve Prolapse and Healthy Peers: Findings from Serial Cardiopulmonary Exercise Testing. J Cardiovasc Dev Dis. 2023 Apr 13;10(4):167. doi: 10.3390/jcdd10040167. PMID: 37103046; PMCID: PMC10141154.
16. Sonaglioni A, Nicolosi GL, Lombardo M. The relationship between mitral valve prolapse and thoracic skeletal abnormalities in clinical practice: a systematic review. J Cardiovasc Med (Hagerstown). 2024 May 1;25(5):353-363. doi: 10.2459/JCM.0000000000001614. Epub 2024 Mar 19. PMID: 38526955.
17. Sonaglioni A, Baravelli M, Vincenti A, Trevisan R, Zompatori M, Nicolosi GL, et al. A New modified anthropometric haller index obtained without radiological exposure. Int J Cardiovasc Imaging. 2018;34(10):1505-1509.
18. Archer JE, Gardner A, Berryman F, Pynsent P. The measurement of the normal thorax using the Haller index methodology at multiple vertebral levels. J Anat. 2016;229(4):577-81.
19. Sonaglioni A, Nicolosi GL, Lombardo M, Gensini GF, Ambrosio G. Influence of chest conformation on myocardial strain parameters in healthy subjects with mitral valve prolapse. Int J Cardiovasc Imaging. 2021 Mar;37(3):1009-1022. doi: 10.1007/s10554-020-02085-z. Epub 2020 Oct 30. PMID: 33128156.

20. Sonaglioni A, Nicolosi GL, Rigamonti E, Lombardo M. Impact of Chest Wall Conformation on the Outcome of Primary Mitral Regurgitation due to Mitral Valve Prolapse. J Cardiovasc Echogr. 2022 Jan-Mar;32(1):29-37. doi: 10.4103/jcecho.jcecho_71_21. Epub 2022 Apr 20. PMID: 35669134; PMCID: PMC9164916.
21. Pu M, Prior DL, Fan X, Asher CR, Vasquez C, Griffin BP, Thomas JD. Calculation of mitral regurgitant orifice area with use of a simplified proximal convergence method: initial clinical application. J Am Soc Echocardiogr. 2001 Mar;14(3):180-5. doi: 10.1067/mje.2001.110139. PMID: 11241013.
22. Nishimura RA, Otto CM, Bonow RO, Carabello BA, Erwin JP 3rd, Fleisher LA, Jneid H, Mack MJ, McLeod CJ, O'Gara PT, Rigolin VH, Sundt TM 3rd, Thompson A. 2017 AHA/ACC Focused Update of the 2014 AHA/ACC Guideline for the Management of Patients With Valvular Heart Disease: A Report of the American College of Cardiology/American Heart Association Task Force on Clinical Practice Guidelines. Circulation. 2017 Jun 20;135(25):e1159-e1195. doi: 10.1161/CIR.0000000000000503. Epub 2017 Mar 15. PMID: 28298458.
23. Galderisi M, Cosyns B, Edvardsen T, Cardim N, Delgado V, Di Salvo G, Donal E, Sade LE, Ernande L, Garbi M, Grapsa J, Hagendorff A, Kamp O, Magne J, Santoro C, Stefanidis A, Lancellotti P, Popescu B, Habib G; 2016–2018 EACVI Scientific Documents Committee; 2016–2018 EACVI Scientific Documents Committee. Standardization of adult transthoracic echocardiography reporting in agreement with recent chamber quantification, diastolic function, and heart valve disease recommendations: an expert consensus document of the European Association of Cardiovascular Imaging. Eur Heart J Cardiovasc Imaging. 2017 Dec 1;18(12):1301-1310. doi: 10.1093/ehjci/jex244. PMID: 29045589.
24. Humbert M, Kovacs G, Hoeper MM, Badagliacca R, Berger RMF, Brida M, Carlsen J, Coats AJS, Escribano-Subias P, Ferrari P, Ferreira DS, Ghofrani HA, Giannakoulas G, Kiely DG, Mayer E, Meszaros G, Nagavci B, Olsson KM, Pepke-Zaba J, Quint JK, Rådegran G, Simonneau G, Sitbon O, Tonia T, Toshner M, Vachiery JL, Vonk Noordegraaf A, Delcroix M, Rosenkranz S; ESC/ERS Scientific Document Group. 2022 ESC/ERS Guidelines for the diagnosis and treatment of pulmonary hypertension. Eur Heart J. 2022 Oct 11;43(38):3618-3731. doi: 10.1093/eurheartj/ehac237. Erratum in: Eur Heart J. 2023 Apr 17;44(15):1312. doi: 10.1093/eurheartj/ehad005. PMID: 36017548.
25. Lang RM, Badano LP, Mor-Avi V, Afilalo J, Armstrong A, Ernande L, Flachskampf FA, Foster E, Goldstein SA, Kuznetsova T, Lancellotti P, Muraru D, Picard MH, Rietzschel ER, Rudski L, Spencer KT, Tsang W, Voigt JU. Recommendations for cardiac chamber quantification by echocardiography in adults: an update from the American Society of Echocardiography and the European Association of Cardiovascular Imaging. J Am Soc Echocardiogr. 2015 Jan;28(1):1-39.e14. doi: 10.1016/j.echo.2014.10.003. PMID: 25559473.
26. Nagueh SF, Smiseth OA, Appleton CP, Byrd BF 3rd, Dokainish H, Edvardsen T, Flachskampf FA, Gillebert TC, Klein AL, Lancellotti P, Marino P, Oh JK, Popescu BA, Waggoner AD. Recommendations for the Evaluation of Left Ventricular Diastolic Function by Echocardiography: An Update from the American Society of Echocardiography and the European Association of Cardiovascular Imaging. J Am

Soc Echocardiogr. 2016 Apr;29(4):277-314. doi: 10.1016/j.echo.2016.01.011. PMID: 27037982.
27. Voigt JU, Pedrizzetti G, Lysyansky P, Marwick TH, Houle H, Baumann R, Pedri S, Ito Y, Abe Y, Metz S, Song JH, Hamilton J, Sengupta PP, Kolias TJ, d'Hooge J, Aurigemma GP, Thomas JD, Badano LP. Definitions for a common standard for 2D speckle tracking echocardiography: consensus document of the EACVI/ASE/Industry Task Force to standardize deformation imaging. Eur Heart J Cardiovasc Imaging. 2015 Jan;16(1):1-11. doi: 10.1093/ehjci/jeu184. Epub 2014 Dec 18. PMID: 25525063.
28. Pellikka PA, Arruda-Olson A, Chaudhry FA, Chen MH, Marshall JE, Porter TR, Sawada SG. Guidelines for Performance, Interpretation, and Application of Stress Echocardiography in Ischemic Heart Disease: From the American Society of Echocardiography. J Am Soc Echocardiogr. 2020 Jan;33(1):1-41.e8. doi: 10.1016/j.echo.2019.07.001. Epub 2019 Nov 15. PMID: 31740370.
29. Lancellotti P, Troisfontaines P, Toussaint AC, Pierard LA. Prognostic importance of exercise-induced changes in mitral regurgitation in patients with chronic ischemic left ventricular dysfunction. Circulation. 2003 Oct 7;108(14):1713-7. doi: 10.1161/01.CIR.0000087599.49332.05. Epub 2003 Sep 15. PMID: 12975251.
