# Supplementary material for: When Exercise ECG and Stress Echocardiography Disagree: Thoracic Geometry as a Potential Determinant of Exercise-Induced ST-Segment Abnormalities
Source: J Clin Med. 2026 Oct 24;15(21):8292. doi: 10.3390/jcm15218292 (PMC13763842; doi:10.3390/jcm15218292)
Supplement: Supplementary file 1 [file jcm-15-08292-s001.zip › Supplementary Material_File S2.pdf]

03/11/2025

In riferimento allo studio **MVP&ESE** , avente per titolo:

Influenza Della Conformazione Toracica Sulla Tolleranza Allo Sforzo In Una Coorte Prospettica Di Individui Sintomatici Con Prolasso Valvolare Mitralico Sottoposti Ad Ecocardiogramma Da Sforzo Fisico

cui è condizionato il Parere Etico espresso nella seduta del 21.10.2025. si rivaluta la seguente documentazione pervenuta in data 29.10.2025 :

Lettera di trasmissione Lettera-Risposta-CE-Studio_MVP-ESE_SONAGLIONI.pdf Lettera Risposta CE
Informativa e consenso base 03_Foglio-Informativo-e-Consenso-con-privacy_-MVP-ESE_V1.5_25.05.2025_CL.pdf 03_Foglio-Informativo-e-Consenso-con-privacy_-MVP-ESE_V1.5_25.05.2025_TC.pdf Foglio Informativo e Consenso con privacy TC e clean

Presa visione della documentazione. che recepisce le richieste di modifica avanzate del nostro CET Lombardia 5, viene sciolta la riserva.

Il Segretario
Dott. Michele Tedeschi

Firmato digitalmente da:
Michele Tedeschi
Data: 03/11/2025 11:21:46
